# Chiral Thioureas—Preparation and Significance in Asymmetric Synthesis and Medicinal Chemistry

**DOI:** 10.3390/molecules25020401

**Published:** 2020-01-18

**Authors:** Franz Steppeler, Dominika Iwan, Elżbieta Wojaczyńska, Jacek Wojaczyński

**Affiliations:** 1 Faculty of Chemistry, Wrocław University of Science and Technology, Wybrzeże Wyspiańskiego 27, 50 370 Wrocław, Poland; franz.steppeler@pwr.edu.pl (F.S.); dominika.iwan@pwr.edu.pl (D.I.); 2 Faculty of Chemistry, University of Wrocław, 14 F. Joliot-Curie St., 50 383 Wrocław, Poland; jacek.wojaczynski@chem.uni.wroc.pl

**Keywords:** asymmetric synthesis, chirality, isothiocyanates, organocatalysis, stereoselectivity, thioureas

## Abstract

For almost 20 years, thioureas have been experiencing a renaissance of interest with the emerged development of asymmetric organocatalysts. Due to their relatively high acidity and strong hydrogen bond donor capability, they differ significantly from ureas and offer, appropriately modified, great potential as organocatalysts, chelators, drug candidates, etc. The review focuses on the family of chiral thioureas, presenting an overview of the current state of knowledge on their synthesis and selected applications in stereoselective synthesis and drug development.

## 1. Introduction

The replacement of the electronegative oxygen atom of urea by sulfur (with electronegativity comparable to carbon) results in a significant change of properties. Thioureas (thiocarbamides) exhibit higher acidity and are stronger hydrogen bond donors [1,2,3]. This ability to participate in hydrogen bonding, which can be further modified by the appropriate substitution of nitrogen atoms, is essential for numerous applications of this class of organic compounds, mainly in organocatalysis and molecular recognition. Thioureas are also widely applied in agriculture and medicine [4,5,6,7], and as corrosion inhibitors [8,9,10,11]. For almost 20 years they have been used as catalysts in organic synthesis, especially in stereoselective reactions [12,13,14]. They also serve as valuable starting materials for the synthesis of heterocycles [15,16,17], and are used as ligands in coordination chemistry (particularly when additional donors are present in their molecules) [18,19,20,21,22], as well as in the field of anion binding and recognition [2,23].

**Figure 1:**
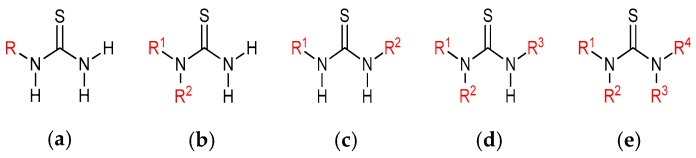
Types of thioureas; (**a**) monosubstituted; (**b**) 1,1-disubstituted; (**c**) 1,3-disubstituted; (**d**) trisubstituted; (**e**) tetrasubstituted.

Structurally thioureas can be classified depending on the number of substituents of nitrogen atom (Figure 1) [6,7]. Not surprisingly, derivatives with one and two organic groups (either 1,1 or 1,3-disubstituted) are most common, though trisubstituted (with limited, but still preserved possibility to act as hydrogen bond donors) and fully substituted (mainly cyclic) thioureas are also prepared and used for various purposes. The first thiocarbamides were prepared ca. 150 years ago [24,25], and their chiral derivatives have been long known [26,27], but a great interest in the latter has arisen with the development of enantioselective organocatalysis.

## 2. Synthesis of Chiral Thioureas

Since the thiourea skeleton is not intrinsically chiral, the existence of enantiomers typically originates from its substituents. Examples of various groups bearing stereogenic centers will be presented in the subsequent section. Desymmetrisation resulting from less common axial (biphenyl or binaphthyl derivatives) [28,29,30] or planar chirality [31,32,33] is also worth mentioning. Consequently, the methods of synthesis of chiral thioureas are essentially the same as for their achiral counterparts, but, typically, the reactants from a chiral pool are used in enantiomerically pure form. However, the preparation of the desired product as racemic mixture followed by separation of enantiomers was reported in selected cases, e.g., bis-thiourea derivatives of Tröger’s base were obtained as racemates and resolved on a chiral stationary phase [34]. Similarly, enantiomers of norbornane thiocarbamide formed as 1:1 mixture in a two-step protocol from norbornene were separated either by chiral HPLC or by crystallization of diastereomeric salts of the amine intermediates [35]. The advantage of this approach lays in the fact that both optical antipodes of chiral thiourea can be isolated and used as organocatalysts or for chiral recognition.

The choice of a particular method of preparation of the desired stereoisomer depends mainly on its structure (number and type of substituents) and availability of necessary starting materials. Certainly, one should also consider possible inconveniences (both for the person conducting the synthesis and for the environment) connected with the use of certain reactants: their limited stability, toxicity, flammability, or simply an unpleasant odor. In most preparations, reactants containing C=S bond are used—mainly isothiocyanates, but also dithiocarbamates, carbon disulfide, thiophosgene and their equivalents. Less frequently, inorganic sources of sulfur are useful for a given transformation—P_4_S_10_ or Lawesson’s reagent to convert urea into thiourea [36,37] sulfides or elemental sulfur. Amines are the most usual source of thiourea nitrogen atoms; however, isocyanides and azides are also found in various protocols. An alternative route involves modification of already constructed achiral thiourea with optically active groups.

### 2.1. Reaction of Isothiocyanates with Amines

**Scheme 1:**
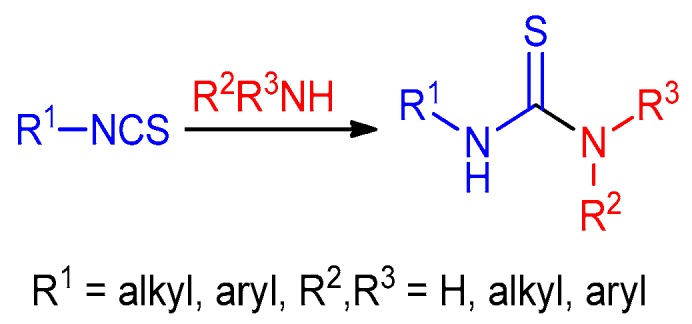
Preparation of thioureas from isothiocyanates and amines or ammonia.

For many years, the most common method of preparation of thiourea, including chiral derivatives, has been based on the reaction of alkyl or aryl isothiocyanate with amine or ammonia (Scheme 1) [1]. This way, unsymmetrical mono-, di- or trisubstituted products are formed, depending on the amine (which can be either aliphatic or aromatic). This substrate typically serves as a source of chirality in most preparations of chiral thioureas, while the isocyanate is chosen to enhance the desired properties of the designed catalyst, ligand, or receptor (most frequently trifluoromethyl-substituted aryl derivatives are used). However, chiral isothiocynates are used as well, as they can be easily obtained from the respective primary amine and CS_2_ [38].

The method is versatile and convenient from the point of view of atom economy. Only one C–N bond has to be formed in the course of the reaction. Among drawbacks, limited long-term stability of isothiocyanate reactants and the possibility of formation of side products (e.g., uretanes in case when alcohols are used as solvents) are often mentioned.

**Scheme 2:**
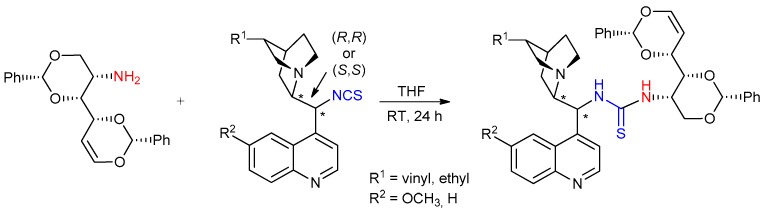
Chiral thiourea organocatalysts prepared from d-mannitol derivative and *Cinchona* alkaloids [40].

Recent examples of the application of this route to synthesize enantiomerically pure thioureas include the paper by Sureshbabu et al., who prepared 25 new *N*-urethane-protected amino alkyl isothiocyanates [39]. A CS_2_/trimethylamine/tosyl chloride combination was found to be the most efficient for the conversion of mono-*N*-protected chiral diamines, which in turn were obtained from the corresponding amino acids. The coupling of the isothiocyanates with amino acid esters in the presence of *N*,*N*-diisopropylethylamine resulted in formation of dithioureidopeptides in 65–74% yield. Liu et al. described a preparation of five new chiral thiourea organocatalysts using amines obtained in four steps from d-mannitol and isocyanates (mainly those derived from *Cinchona* alkaloids, Scheme 2) [40]. Isothiocyanates were also reacted with sulfoximines [41] and phosphoramides [42] to give the respective thioureas.

**Scheme 3:**
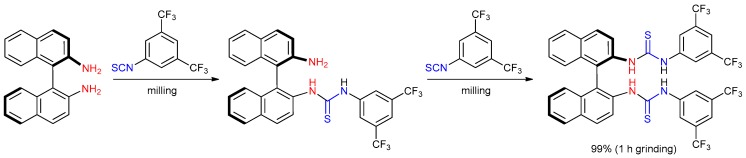
Mechanochemical synthesis of axially chiral thiourea [47].

Modifications of typical reaction conditions have been recently proposed. Possible application of solvent-free protocols, with reactants in a gas- and/or solid state, and mechanochemical synthesis were explored [43,44,45,46]. As an example, neat grinding and liquid-assisted grinding were applied to the preparation of six (already known) chiral bis-thioureas in quantitative yields by Štrukil et al. (Scheme 3) [47]. Bhattacharjee et al. demonstrated the utility of phosphinoselenoicamide-supported titanium(IV) complex as a pre-catalyst for the addition of amines to carbodiimides, isocyanates, and isothiocyanates [48]. 1,3-Disubstituted derivatives were prepared in 75–99% yield. Arafa et al. reported on the ultrasound-assisted synthesis of bis-thioureas from isocyanates and diamines; medium and high reaction yield were observed after several minutes of sonication [49].

Methods involving inorganic isothiocyanates are less commonly used, but can be treated as an interesting option when the required organic isothiocyanate is not available. Herr and co-workers showed that monosubstituted and symmetrical 1,3-disubstituted thioureas can be prepared in high yields from amine hydrohalides (including chiral ones) and KSCN [50]. Once prepared thiourea can be easily modified as discussed in Section 2.5, to achieve the necessary substitution pattern.

An interesting example of stereoselective preparation of chiral cyclic thioureas in the reaction of organic isothiocyanates with *N*-tosylimines was demonstrated by Wang and co-workers [51]. The reaction was efficiently catalyzed by another chiral, rosin-derived thiourea (see Section 3.7).

### 2.2. Reaction of Amines with Dithiocarbamates

**Scheme 4:**
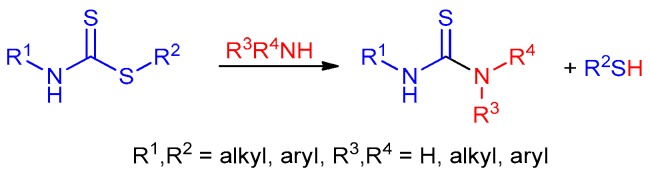
Preparation of thioureas from diacarbamates and amines.

Preparation of thioureas from dithiocarbamate derivatives and amines (Scheme 4) can serve as alternative to the route involving isothiocyanates (which are, in fact, formed in the course of the reaction). However, only in certain protocols isolated, stable dithiocarbamates are used, and in most cases, they are prepared in situ from CS_2_ and amines (see Section 2.3). In the recent years, several reports of efficient preparations of diverse dithiocarbamates have been published. A reaction in water involving also α,β-unsaturated compounds, and solvent- and catalyst-free protocol with alkyl halides both furnished appropriately substituted dithiocarbamates as reported by Azizi et al. [52,53]. Similarly, hydroxylated derivatives were prepared by Ziyaei-Halimjani and Saidi from amines, CS_2_ and epoxides [54]. Vinyl pyridines and vinyl pyrazine served as Michael acceptors in another protocol for efficient synthesis of dithiocarbamates [55]. In turn, the use of DEAD or DIAD resulted in products bearing a sulfur-nitrogen bond, which were found to be efficient intermediates in the synthesis of thioureas, isothiocyanates and thiocarbamates [56].

The majority of publications concerning transformations of dithiocarbamates into thioureas focus on the synthesis of achiral derivatives. For example, various di- and trisubstituted thioureas were obtained in 63–92% yield via the reaction of trimethylamine or DABCO salts of dithiocarbamates and primary or secondary amines [57]. Cerium ammonium nitrate was used as a catalyst, and the condensation was carried out in acetonitrile at room temperature for 2–24 h. A series of 1-aryl-3,3-dimethylthioureas were prepared in 70–92% yield from aryl amines which were first treated with sodium hydride in DMSO and then heated with *S*-aryl-*N*,*N*-dimethyldithiocarbamates for 3–5 h at 90 °C, with the release of thiophenol byproduct [58].

**Scheme 5:**
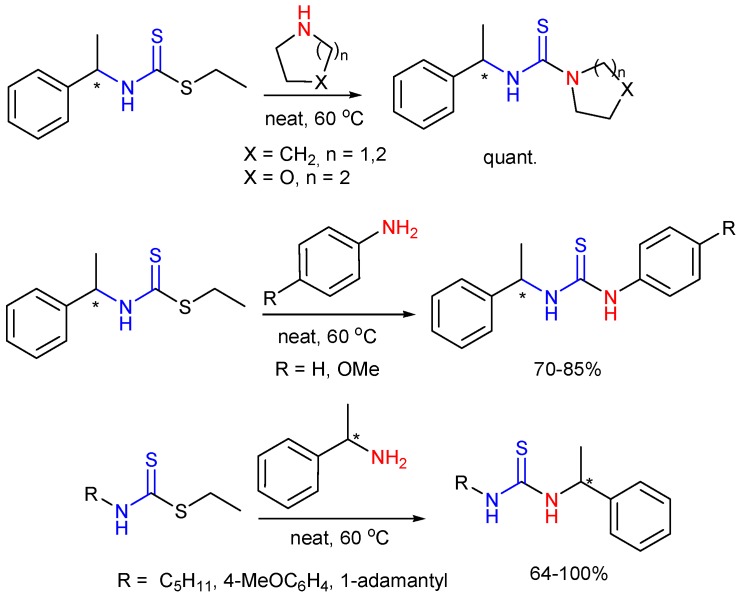
Solvent-free synthesis of non-racemic thioureas from dithiocarbamates and amines [60].

Recently published methods show that the reaction can be performed in water under mild conditions. For example, thiazolidine-2-thiones and secondary amines stirred in aqueous solution at 80 °C for 3–5 h without a catalyst yielded trisubstituted thioureas in 60–90% yield [59]. In another preparation, unsymmetrical thioureas were formed when primary or secondary amines, either aliphatic or aromatic, were heated with dithiocarbamates (derived from primary amines) at 50–60 °C and under solvent-free conditions (Scheme 5) [60]. Yields ranged from 64% to 100%. Among various derivatives obtained, three inherited chirality from the amine, and five from the dithiocarbamate.

**Scheme 6:**
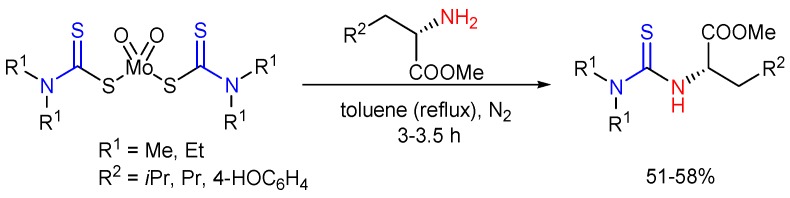
The use of coordinated dithiocarabamate in the preparation of chiral thiourea [62].

The reaction can be also mediated by metals that are complexed with dithiocarbamate ligands. Dirksen et al. reported on the preparation of a mixture of 1,3-disubstituted and trisubstituted thioureas from bis(dimethyldithiocarbamato) zinc(II) and primary amines [61]. Maddani and Prabhu used dioxomolybdenum dialkyl dithiocarbamates and primary amines to prepare eleven thioureas in 51–85% yield (Scheme 6) [62]. The reaction was conducted in refluxing toluene under nitrogen for 0.5–3 h. Four chiral derivatives were prepared starting from methyl esters of l-phenylalanine, l-tyrosine, and l-leucine.

The formation of thioureas upon self-condensation of trialkylammonium dithiocarbamates was reported as well [63].

### 2.3. The Use of Carbon Disulfide

**Scheme 7:**
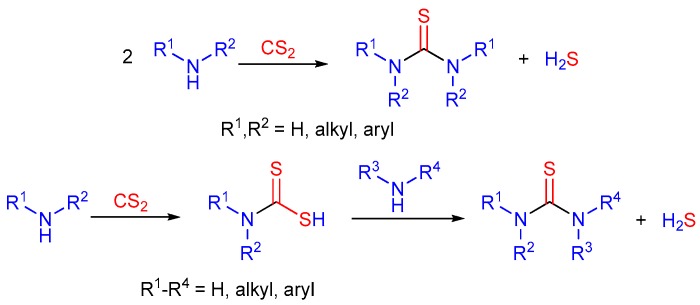
Synthesis of symmetrical and unsymmetrical thioureas from carbon disulfide and amines.

Reactions of amines with carbon disulfide serve as a simple method for the preparation of symmetrical, 1,3-disubstituted thioureas. Using a modified protocol with two different amines opens the route to unsymmetrical, mono-, di- or trisubstituted products (Scheme 7). Transient formation of dithiocarbamates and isothiocyanates was suggested as a key step in the mechanism of the process [1]. Like other C=S transfer reagents, carbon disulfide is not free of drawbacks: it is flammable, volatile (which requires an excess to be used), and has an unpleasant smell. Atom economy suffers from the formation of sulfur-containing by-product, usually hydrogen sulfide. The reaction is typically performed in organic solvents at elevated temperature and is relatively slow; it can be accelerated by addition of bases or oxidants to remove H_2_S [1]. Furthermore, CBr_4_ was shown by Liang et al. to efficiently promote the reaction [64].

**Scheme 8:**
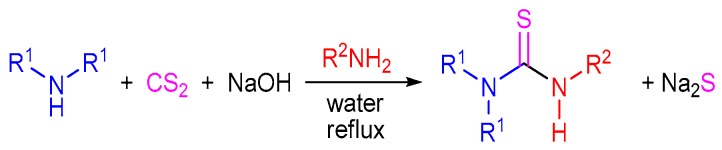
Preparation of thioureas in refluxing water [66].

**Scheme 9:**
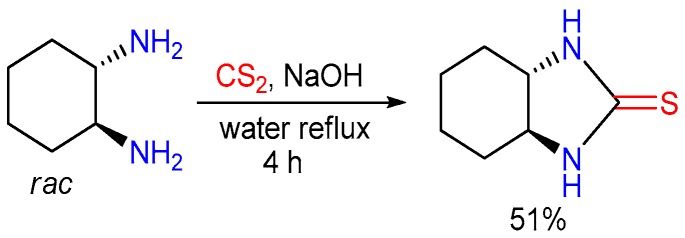
Synthesis of a chiral, racemic thiourea from diamine and CS_2_ [66].

Most recent modifications involve the use of water, a convenient and green solvent, as reaction medium [65]. A simple condensation between amines and carbon disulfide in refluxing water was described by Maddani and Prabhu as a route to symmetrical and unsymmetrical di- and trisubstituted thiourea derivatives [66]. The method worked well with aliphatic amines: in the first step, a secondary or primary amine was treated with CS_2_ in aqueous NaOH under ambient conditions and thus prepared dithiocarbamates were heated under reflux with primary amines for 3–12 h; after acidic work-up the desired thioureas were isolated in good to high yields (19 examples, 40–93%, Scheme 8). The use of racemic 1-phenylethylamine and (1*R*,2*R*)-1,2-diaminocyclohexane illustrates the possibility of using the protocol to the synthesis of chiral derivatives (Scheme 9).

**Scheme 10:**
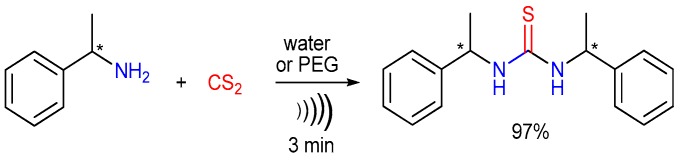
Ultrasound-assisted synthesis of chiral thioureas [68].

Two chiral and twenty-three achiral symmetrical disubstituted thioureas were prepared in 70–97% yield by Azizi et al. from various primary amines and CS_2_; the reaction was carried out in water at 60 °C for 1–12 h (aromatic amines required a longer reaction time) and purification involved only filtration, washing with water and recrystallization from ethanol or diethyl ether [67]. Six years later, the same group described an ultrasound-assisted synthesis of 1,3-disubstituted, but also trisubstituted thiourea derivatives (Scheme 10) [68]. The reaction was performed in water or polyethylene glycol, and in the latter yields were higher in most cases (60–97% after 3–6 min of sonication at 30–35 °C). Among 27 products, two enantiomers of 1,3-bis(1-phenylethyl)thiourea were prepared, both in 97% yield.

Seventeen *N-*sulfonylcyclothioureas, including 1 chiral derivative, were prepared in 60–87% yield by Wan et al. in the reaction of corresponding amines with carbon disulfide in water catalyzed by silica [69]. An efficient (90–99% yield), green (water used as solvent), catalyst- and chromatography-free protocol for symmetrical, bis-aliphatic thioureas was also developed by Jangale et al. [70]. Milosavljević et al. described the environmentally friendly methodology involving the use of oxidants (sodium percarbonate + EDTA system was most efficient, but also H_2_O_2_ and air were tested) and solvent recycling [71]. Other modifications of reaction conditions for the reaction of amines with CS_2_ have been proposed, including green variants: the use of solar energy [72], microwaves [73,74], ionic liquid media [75,76].

**Scheme 11:**
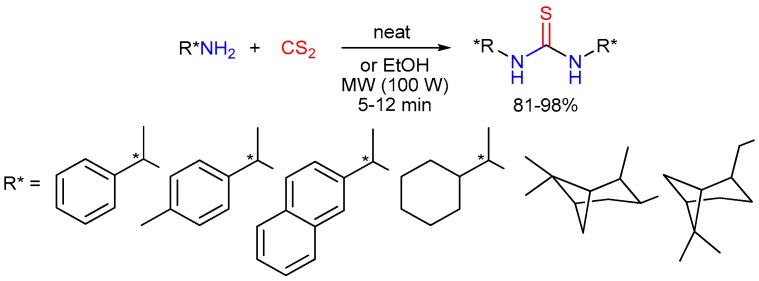
Preparation of chiral, symmetrical thioureas from CS_2_ and amines [77].

Ten chiral thioureas were prepared by Vázquez et al. in the reaction of enantiopure primary amines (including four pairs of enantiomers) with carbon disulfide under either solvent-free conditions or using microwave irradiation in ethanol (Scheme 11) [77]. Simple mixing of liquid reactants resulted in immediate and high yielding (91–97%) reaction, though product isolation and purification was required. Slightly lower yields (81–95%) were noted for 5 min of MW irradiation, however, pure products crystallized from ethanol used as solvent. Conventional heating was found time-consuming and less efficient (75–88% yield).

**Scheme 12:**
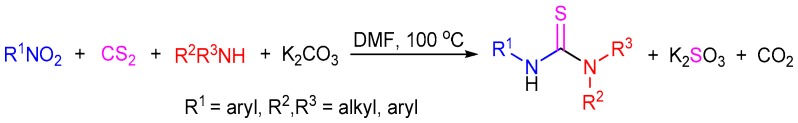
Preparation of thioureas from nitro compounds, carbon disulfide and amines [78].

**Scheme 13:**
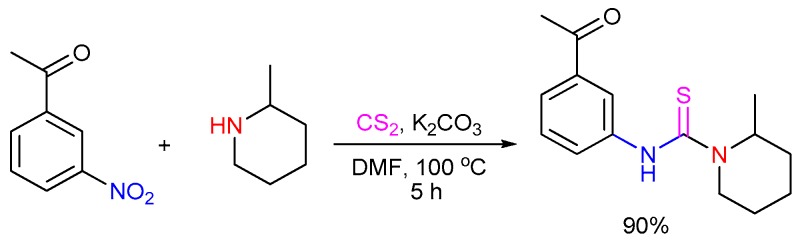
A racemic thiourea prepared by Dutta et al [78].

In 2019, a route to unsymmetrical thioureas was proposed by Dutta et al. involving the reaction of dithiocarbamate anions, generated in situ from secondary or primary amines and carbon disulfide at low temperature, with aromatic nitro compounds (Scheme 12) [78]. DMF as solvent, potassium carbonate as a base, and temperature of 100 °C were established as optimal conditions for the second step of the reaction. Among 22 derivatives obtained in 77–93% yield after 4–6 h one was chiral, but apparently racemic (Scheme 13). The postulated reaction mechanism involved the formation of nitrosoaryl intermediate and release of SO_2_ (consumed by K_2_CO_3_) as a by-product resulting from dithiocarbamate oxidation by nitroarene.

**Scheme 14:**
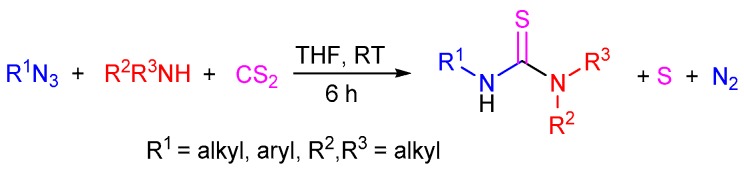
Azides in the syntheisis of thioureas [79].

Organic azides were also converted to the corresponding thioureas. Kumar et al. reported on the synthesis of thioureido peptidomimetics using *N*-protected amino alkyl azides and dithiocarbamoic acids formed in situ from carbon disulfide and primary or secondary amines (Scheme 14) [79]. The reaction proceeded with the liberation of N_2_ and sulfur; it was performed in THF in the presence of pyridine at 0 °C to room temperature under inert atmosphere for 6 h. Fifteen enantiomerically pure derivatives were isolated in 72–85% yield.

### 2.4. Application of Other Compounds Containing C=S Bond

**Scheme 15:**
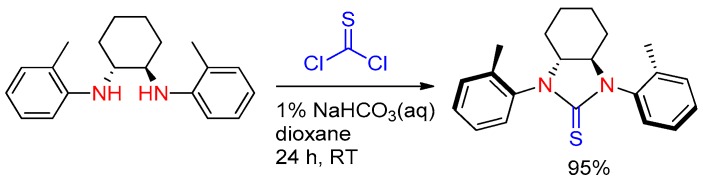
The use of thiophosgene in the preparation of a chiral cyclic thiourea [81].

Less frequently, other thioorganic compounds find their application in the synthesis of thioureas, both chiral and achiral derivatives. In the past, a reaction of thiophosgene with primary or secondary amines was used in such preparations, though this source of C=S fragment has not gained popularity due to its corrosive and toxic properties [80]. However, its reaction with diamines was a key step in the synthesis of chiral cyclic thiourea ligands for palladium-catalyzed reactions by Yang and co-workers (Scheme 15) [81,82]. 1-(Methyldithiocarbonyl)imidazole and its quaternary *N-*methylated salt were found by Mohanta et al. to be effective thiocarbonyl transfer agents which allowed preparation of mono-, di- and trisubstituted thioureas (though not chiral) under mild conditions [83].

**Scheme 16:**
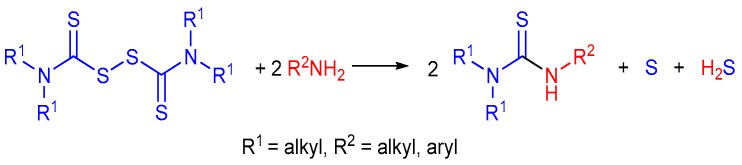
Thiuram disulfide as a reactant in preparation of thioureas [84].

**Scheme 17:**
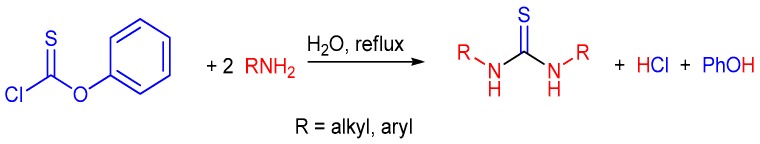
Synthesis of symmetrical thioureas from phenyl chlorothionoformate and amine [85].

**Scheme 18:**
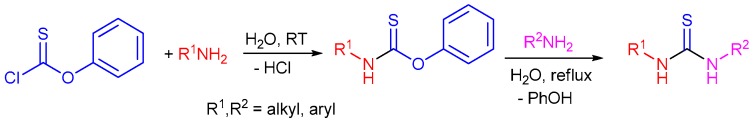
Two-step approach to unsymmetrical thioureas from phenyl chlorothionoformate [86].

Furthermore, achiral derivatives were prepared in the reaction of amines with thiuram disulfides (Scheme 16) or monosulfides (in turn obtained from dithiocarbamates) [84]. Li et al. developed a protocol utilizing phenyl chlorothionoformate [85,86]. Its reaction with primary amines (either aliphatic or aromatic) in refluxing water carried out for 2–12 h (though 2–3 h were sufficient in most cases) afforded a series of 17 symmetrical, 1,3-disubstituted thioureas in 60–99% yield (Scheme 17) [85]. Among them, two enantiomerically pure derivatives were prepared. The protocol was also used to obtain thione heterocycles, however, it failed in the attempts with a mixture of two amines aimed to yield unsymmetrically substituted products. Such compounds could be formed using a two-step route: amines were first reacted with chloroformate in water for 1 h at room temperature [86]. Then, the resulting thiocarbamate was converted into the desired thiourea by heating with another (more reactive) amine for 20–80 min at 100 °C (Scheme 18). The yields were high (87–95% for the first step, 75–97% for the second one), products (none of them were chiral) were isolated by filtration and washed with water, without the need of further purification. The reaction could be performed on a gram scale.

### 2.5. Preparation of Chiral Thioureas from Other Thioureas

**Scheme 19:**
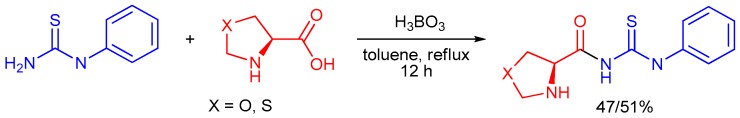
Reaction of monosubstituted thiourea with a chiral carboxylic acid as a route to a chiral thiourea [87].

The thiocarbonyl moiety of chiral thiourea can also originate from achiral ones, subjected to an appropriate modification. In principle, it is possible to preserve the original skeleton and replace groups attached to the nitrogen atom(s), changing the substitution pattern. For example, the thiazolidine motif was introduced via the reaction of mono-aryl-substituted thioureas and the appropriate carboxylic acids with boronic acid catalyst (Scheme 19) [87]. Various prepared *N-*acyl derivatives included chiral ones and were found to exhibit antioxidant activity. Appropriate modifications of substituents of thiourea derived from 1,2-phenylenediamine were described by Liang et al. resulting in the introduction of chirality, and, finally, isolation of atropoisomeric N,S-donating ligands bearing oxazoline moiety [88].

**Scheme 20:**
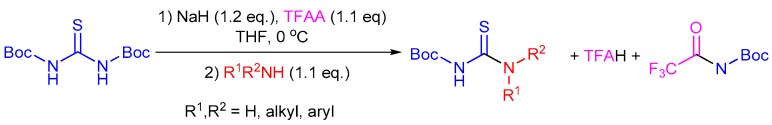
Conversion of bis-Boc thiourea into other thioureas.[89]

Quite frequently, a reaction at thiocarbonyl carbon atom of thiourea is performed, resulting in C–N bond cleavage and attachment of an amine nucleophile. Yin et al. reported on the application of readily available and stable 1,3-bis-Boc-substituted thiourea as thioacylating agent of nucleophiles (amines, but also alcohols, thiols, thiophenolates, sodium malonates, Scheme 20) [89]. Thiocarbonyl compounds were formed in reasonable yields (78–94% in case of thioureas) under mild conditions (sodium hydride as a base, trifluoroacetic acid anhydride (TFAA) as an activator, THF solvent, 0 °C to RT). Amines bearing additional functional groups could be converted into desired products as well, including two enantiomerically pure derivatives for which no epimerization was observed. However, for hindered acyclic secondary amines, the procedure did not work. Bis-Boc thiourea was also utilized by Cohrt and Nielsen in their synthesis of *N*-terminally modified α-thiourea peptides that were further converted into the respective thiazoles, which in turn were incorporated into 15- to 17-membered macrocycles bearing up to three stereocenters [90].

**Scheme 21:**
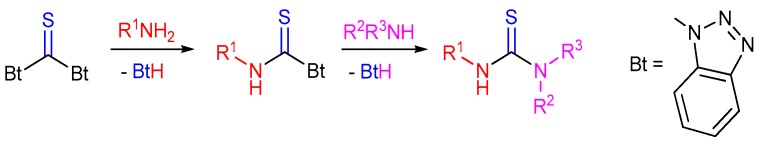
Benzotriazole derivative in the synthesis of appropriately substituted thioureas [91].

**Scheme 22:**
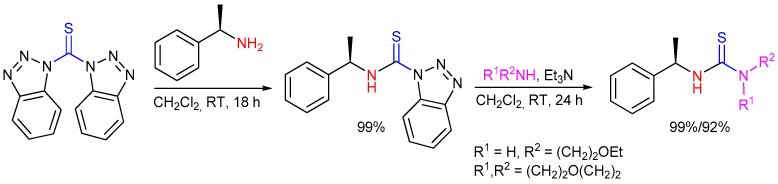
Preparation of chiral thioureas from 1,3-bis-benzotriazole-substituted derivative [91].

**Figure 2:**
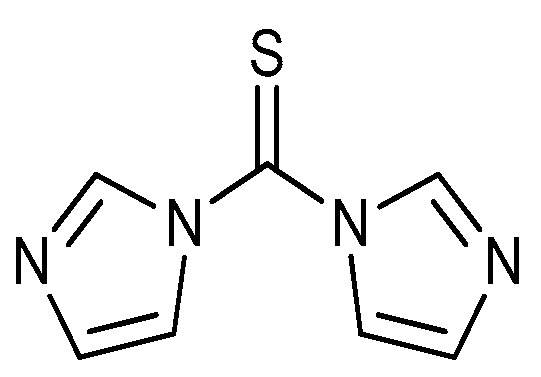
Bis-imidazole thiourea used in the synthesis of other derivatives [92].

Thiourea derivatives bearing heterocyclic substituents were also found to be useful thioacylating agents. 1-(Alkyl/Arylthiocarbamoyl)benzotriazoles were applied by Katritzky et al. as stable isothiocyanate equivalents (Scheme 21) [91]. Among di- and trisubstituted thioureas, three (*R*)-1-phenylethyl derivatives were formed in 92–99% yield (Scheme 22). Kang et al. used 1,1′-thiocarbonyldiimidazole (Figure 2) for C=S transfer, and converted it into desired monosubstituted (achiral) thioureas in two subsequent reactions with primary amines (mainly fluorene derivatives) and NH_3_ [92].

### 2.6. C=S Bond Formation

**Scheme 23:**
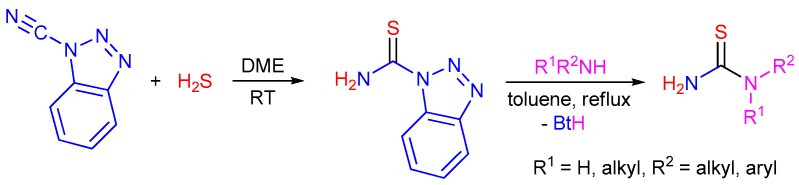
Utilization of hydrogen sulfide in the synthesis of thioureas [93].

**Scheme 24:**
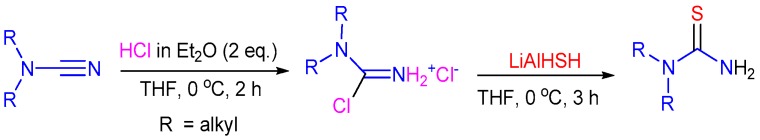
Cyanamide as a starting point for the preparation of thioureas [94].

Less frequently, thioureas have been prepared via multi-component reactions in which elementary sulfur or other sulfur-transfer agents are used to form the C=S bond. Hydrogen sulfide was used by Katritzky et al. as the source of sulfur and reacted with 1-cyanobenzotriazole and amines or with carboximidamides to give thioureas in reasonable to high yields (54–99%, with two exceptions, Scheme 23) [93]. Thioureas (and selenoureas) were also prepared from cyanamides as described by Koketsu et al. [94]. The reaction with HCl in Et_2_O and then with LiAlHSH afforded 1,1-disubstituted products in 52–89% yield (Scheme 24). Unfortunately, chiral derivatives were not prepared by these two groups.

**Scheme 25:**
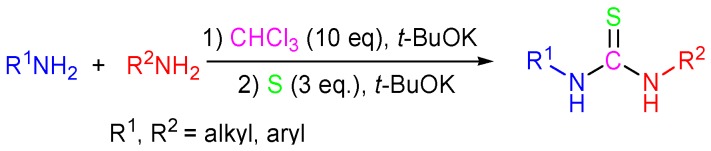
Reaction of amines with sulfur and chloroform as a route to thioureas [95].

Tan et al. described an application of chloroform and elementary sulfur acting as thiocarbonyl surrogate (Scheme 25) [95]. Various primary amines were treated with chloroform in the presence of a base followed by addition of sulfur and a second primary amine. Optimal reaction conditions comprised *t*-BuOK used as a base, *tert*-butanol/1,4-dioxane (1:1 mixture) solvent, 55 °C, and reaction times varied from 6 to 15 h. Yields ranged from 51% to 96%, and among 35 thioureas ten chiral derivatives were obtained with a complete preservation of optical purity of the starting amines.

**Figure 3:**
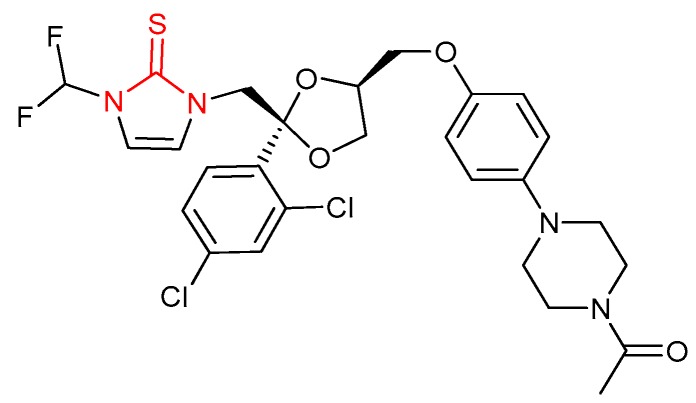
Ketoconazole thiourea derivative prepared by Tang and co-workers [96].

Sulfuration of azoles connected with *N*-difluoromethylation yielding a family of appropriately substituted cyclic thioureas was reported by Tang and co-workers [96]. The optimum reaction conditions were established as 24 h at 100 °C in DMA solvent with sodium hydroxymethylsulfite additive; elementary sulfur and ethyl 2-bromo-2,2-difluoroacetate were used as inexpensive reagents. Two products—derivatives of Econazole (48%) and Ketoconazole (43%)—were chiral, and the latter was obtained as a single enantiomer (Figure 3).

**Scheme 26:**
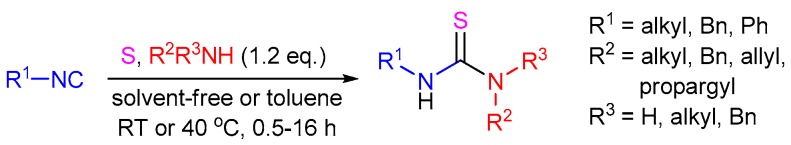
Preparation of thioureas from isocyanides, amines and elementary sulfur [98].

A relatively low explored synthetic strategy is based on the use of isocyanides. Zhu et al. described a Co(II)-catalyzed insertion of isocyanides into active N-H bonds of amines under ultrasound irradiation; the amino methylidyneaminium intermediates reacted readily with various nucleophiles including sulfur (to give thioureas) or water (yielding ureas) [97]. A series of aniline derivatives were tested with *tert*-butyl isocyanide to give 37–53% yield, and tryptamine with 4 different isocyanides gave products in 53–67%. The optimal reaction conditions included 20 mol% of Co(acac)_2_ catalyst, two equivalents of Na_2_CO_3_ and one equivalent of TBHP in 1,4-dioxane and ultrasound irradiation at 75 °C. Chiral thioureas were not prepared, however, two enantiomers of urea were obtained from enantiopure 1-phenylethylamines and *tert*-butyl isocyanide with a complete retention of configuration, suggesting a similar possibility for the synthesis of chiral thiourea. An efficient, three-component reaction of isocyanides, aliphatic amines and elemental sulfur under mild conditions (RT to 40 °C, solvent-free or toluene) was also reported by Nguyen et al. (Scheme 26) [98].

**Scheme 27:**
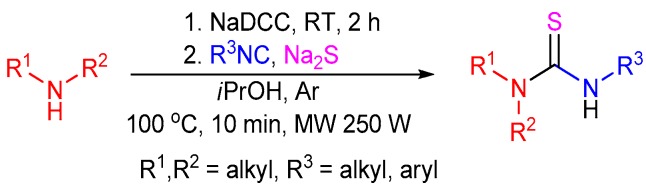
Synthesis of thioureas from amines, isocyanides, and sodium sulfide [99].

**Scheme 28:**
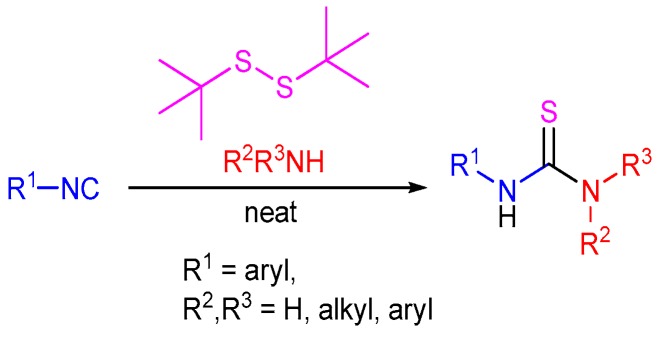
Preparation of thioureas from isocyanides, amines and disulfide [100].

A three-component reaction of isocyanides, in situ formed *N*-chlorinated secondary amines and water or sodium sulfide was reported on by Angyal et al. [99]. Sodium dichloroisocyanurate (NaDCC) was used as a chlorinating agent. The reactions were performed in isopropanol under microwave-assisted conditions (100 °C, 10 min, 250 W, Scheme 27). Eight thioureas were prepared in 27–68% yield, including an enantiopure proline derivative (30%). Singh and Sharma developed a multicomponent protocol of thiourea preparation utilizing aromatic isocyanides, amines and 1,2-di(*tert*-butyl)disulfide under solvent- and catalyst-free conditions (Scheme 28) [100]. Isocyanides were obtained by formylation of aromatic amines followed by dehydration using POCl_3_. All the reactions were heated for 5 h at 120 °C and 1 h at 60 °C after the addition of amines. Twenty-seven thioureas were prepared in 56–99% yield, including an optically active derivative obtained from phenyl isocyanide *Cinchona*mine (57%).

## 3. Types of Chiral Thioureas and Selected Applications in Asymmetric Synthesis

Chiral thioureas can be classified taking into account a moiety introduced to its structure in order to induce chirality. Most structures contain a stereogenic center attached to a nitrogen atom of thiourea which typically derives from amine used as one of the reactants. In many cases, also additional elements of chirality are present, as well as functional groups designed to participate in desired interactions with target molecules (e.g., substrates of catalyzed reaction) or ions (metal ions in coordination compounds, anions bound by thiourea-based receptors). In particular, derivatives based on chiral diamines have attracted attention due to their possible application as bifunctional catalysts, with thiourea moiety acting as hydrogen bond donor and an amino group as a Brønsted base/Lewis base/nucleophile. Alternatively, systems consisting of an achiral thiourea together with components responsible for asymmetric induction are tested as well [101].

**Figure 4:**
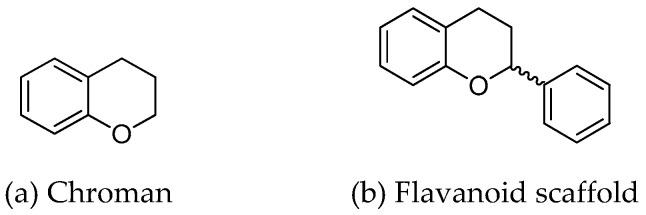
General structures of chroman and flavonoid scaffolds.

Several extensive reviews have been published on asymmetric organocatalysis and, in particular, the use of chiral ureas and thioureas in various stereoselective reactions [3,12,13,14,102,103,104,105,106,107,108,109,110,111,112,113,114]. Consequently, the following part is focused on the presentation of selected examples of compounds with only a short mention about their applications in the synthesis and functionalization of one particular system, i.e., chroman scaffold and its derivatives (Figure 4). As an example for a versatile structure with various pharmaceutically active derivatives [115], it has various possible medicinal usages [116,117]. Prochiral carbon atoms can be substituted, yielding structures with several stereogenic centers, e.g., flavonoids (Figure 4) [118,119]. Preparation of various chroman derivatives with a defined stereochemistry challenged organic chemists. Versatile, general methods for their asymmetric synthesis have been found in recent years [117,120,121,122].

### 3.1. Thioureas Containing trans-1,2-diaminocyclohexane (DACH) Skeleton and Other Chiral Diamines

**Figure 5:**
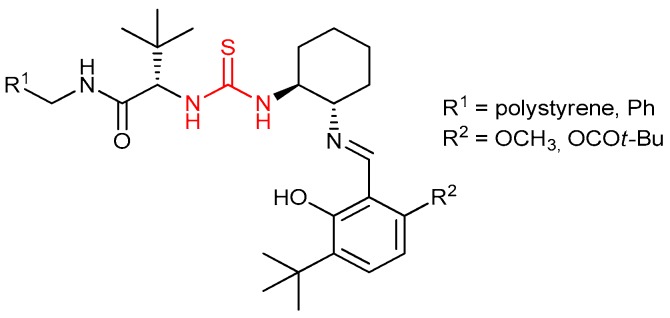
Jacobsen’s thioureas derived from 1,2-diaminocyclohexane [123,124,125].

Pioneering works on application of chiral thioureas in enantioselective organocatalysis came from the laboratory of Jacobsen. Three libraries of polystyrene-supported enantiopure ligands containing amino acid residues, urea or thiourea moiety, and Schiff bases (the last two subunits linked with (*R*,*R*)-1,2-diamines) were evaluated as organocatalysts for asymmetric Strecker reactions (Figure 5) [123,124,125]. The optimized catalyst and its soluble analogues showed a broad substrate scope. Similar, appropriately modified Schiff base catalysts were found optimal in other asymmetric reactions, including hydrophosphonylation of imines [126], intermolecular addition of indoles to cyclic *N*-acyl iminium ions [127], hydro- and acylcyanation of imines [128,129,130]. Furthermore, imidazole derivatives of Schiff bases were applied in Strecker reaction by Tsogoeva et al. [131]. 

**Figure 6:**
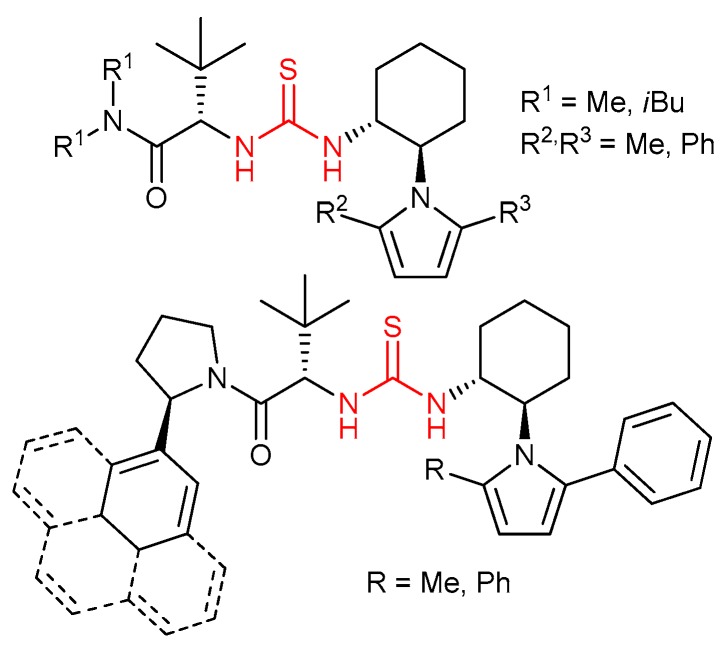
Modified Jacobsen’s organocatalysts.[132,133,134]

Later on, Jacobsen and co-workers developed other DACH-based thiourea organocatalysts (Figure 6) [132,133,134]. For example, pyrrole derivative appeared most efficient in acyl Pictet-Spengler reaction [133], and Cope-type hydroamination [134]. This type of catalyst was also applied by Bui et al. in asymmetric additions of oxoindoles to nitroolefins [135].

**Figure 7:**
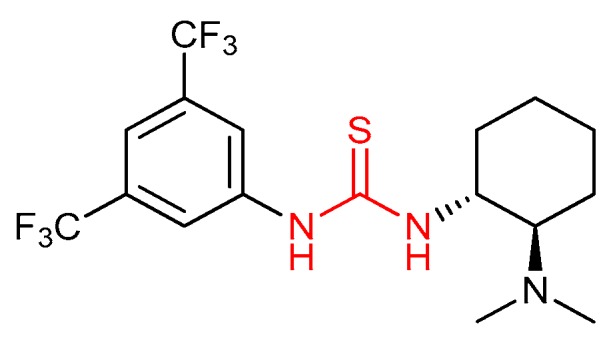
Takemoto’s thiourea organocatalyst [110,111,112].

DACH-based bisfunctional thiourea organocatalysts containing a tertiary amine function were developed by Takemoto and co-workers (Figure 7). A dimethylamine derivative appeared to be a versatile promoter, active in various asymmetric transformations: Michael reaction of nitroolefins and malonates [136] or 1,3-dicarbonyl compounds [137], active methylene compounds with α,β-unsaturated imides [138,139], aza-Henry [140], Neber reaction (synthesis of azirine derivatives) [141], aldol reaction [142], and others [110,111,112].

Now commercially available (in both (*R*,*R*)- and (*S*,*S*) forms), Takemoto’s organocatalyst was also used by other groups, mainly to catalyze various stereoselective additions [135,143,144,145,146,147,148,149,150], and to control the ring-opening polymerization of racemic lactide leading to highly isotactic polylactide [151].

**Figure 8:**
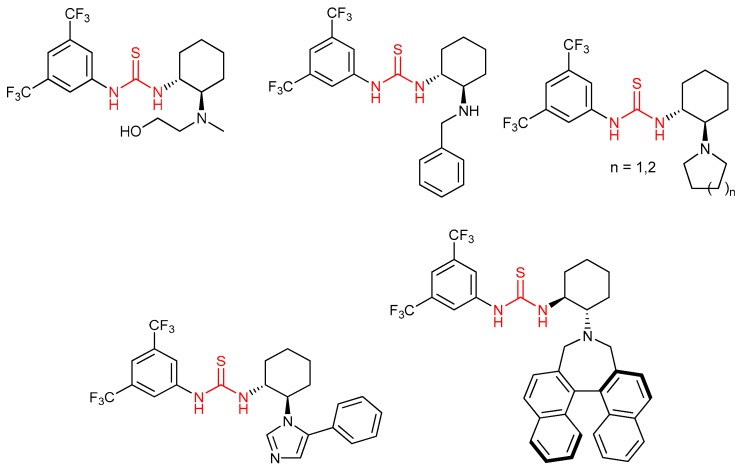
Various chiral DACH-based thioureas used as organocatalysts [153,154,155,156,157,158,159,160,161,162,163].

Diverse modifications of Takemoto’s compound have been introduced, both in the parent group and by others (Figure 8). A PEG-immobilized catalyst was efficient in the enantioselective Michael and tandem Michael reactions [152], while a hydroxylated derivative catalyzed a Petasis-type reaction of quinolones and boronic acids [153]. A hybrid catalyst containing arylboronic acid was evaluated for asymmetric hetero-Michael addition to unsaturated carboxylic acids [154,155]. Various modified derivatives of Takemoto’s catalyst were tested in the enantioselective reduction of ketones with borane; a benzyl-substituted catalyst led to the best outcomes [156]. A piperidine derivative showed the optimal performance among diamine-derived catalysts tested in inverse-electron-demand Diels-Alder cycloaddition [157], Mannich reaction of 2-substituted indolin-3-ones [158], and pyrrolidine-substituted thiourea, in conjugated addition of nitroacetates to unsaturated ketoesters [159], and in double Michael reaction used for the construction of a spiro-fused cyclohexanone-5-oxazolone system [160]. Schreiner and co-workers chose an imidazole-based catalyst (in cooperation with Brønsted acids) as the best one for cyanosilylation of various aldehydes [161]. An axially-chiral binaphthyl-derived amine was used for the synthesis of thioureas which catalyzed stereoselective Michael addition of 2,4-pentanodione and malononitrile to nitroolefins [162,163].

**Figure 9:**
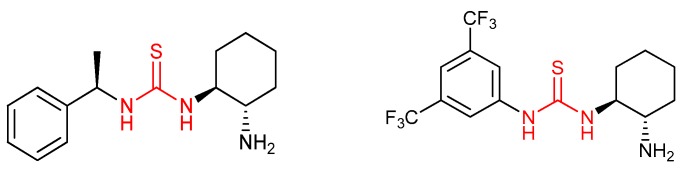
DACH-based thioureas bearing primary amine function [164,165,166,167,168,169,170,171].

A variety of thiourea organocatalysts based on chiral diaminocyclohexane skeleton have been prepared. Selected examples include thioureas bearing primary amine functionalities that were tested in Michael addition of active methylene compounds to enones [164,165,166,167] and of ketones or dioxindoles to nitroolefins (Figure 9) [168,169,170,171].

**Figure 10:**
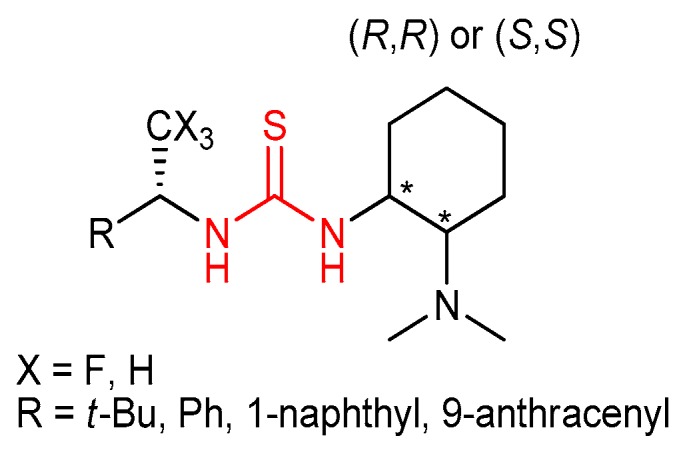
Thiourea derivative with three stereogenic centers [172].

Introduction of additional chiral electron-withdrawing group in place of aryl moiety of Takemoto’s catalyst allowed an additional stereocontrol in Michael addition reaction (Figure 10) [172].

**Figure 11:**
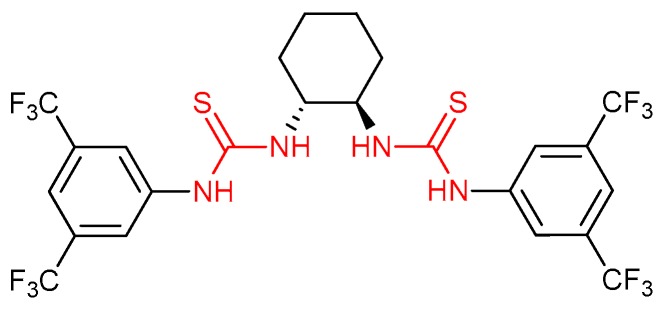
A chiral bis-thiourea prepared by Nagasawa and co-workers [173].

The presence of two amino groups in 1,2-diaminocyclohexane opens the possibility to design and prepare dimeric structures. Nagasawa’s group developed a chiral bis-thiourea organocatalyst efficient in enantioselective Morita-Baylis-Hillman reaction (Figure 11) [173], and its analogues based on other diamines [174]. The DACH-based bis-thiourea was used in other asymmetric transformations, e.g., in photochemical intramolecular [2+2] cycloaddition of 2,3-dihydropiridone-5-carboxylates described by Mayr et al. [175].

**Figure 12:**
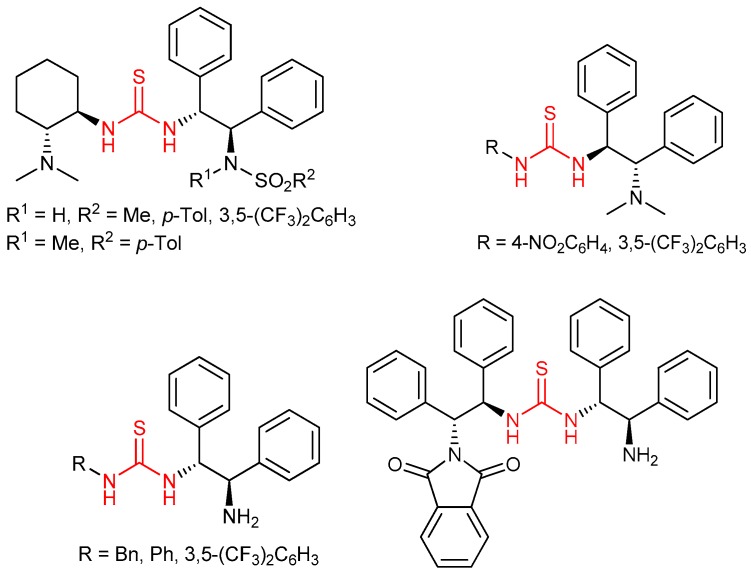
Chiral thioureas based on 1,2-diphenylethylenediamine [176,177,178,179,180,181,182,183].

Other chiral diamines have been converted to thioureas as well. Organocatalysts bearing multiple hydrogen bond donors—chiral diaminocyclohexane and 1,2-diphenylethylenediamine fragments (Figure 12), the latter converted into sulfonamide, were prepared by Wang and co-workers and appeared highly efficient in asymmetric Michael addition as well as nitro-Mannich reaction [176,177]. Among chiral thioureas containing tertiary amines tested in Michael additions of 3-substituted oxindoles to maleimides, and cyanoacetates to vinyl sulfones, 1,2-diphenylethylenediamine derivatives led to the highest yields and stereoselectivities (Figure 12) [178,179]. In turn, primary amines of this kind (Figure 12) were found most efficient in asymmetric additions to unsaturated ketones [180,181] and a stereoselective construction of α,α-disubstituted cycloalkanones [182]. A catalyst bearing two 1,2-diphenylethylenediamine fragments (Figure 12) was used in a tandem asymmetric Michael/cyclization reaction of 4-hydroxycoumarin and nitroalkanes, albeit with moderate yields [183].

**Figure 13:**
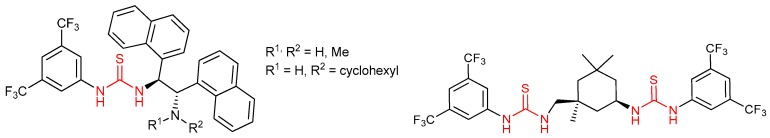
Chiral thioureas preparaed by the groups of Tius (left) [184,185] and Berkessel (right) [186].

1,2-Dinaphthylethylenediamine derivatives were applied by Tius and co-workers in asymmetric Nazorov cyclization of diketones (Figure 13) [184,185]. Berkessel and co-workers converted chiral isophoronediamine into bis-thioureas and bis-ureas (Figure 13) [186]. The derivative containing trifluoromethyl groups appeared the optimum catalyst for asymmetric Morita-Baylis-Hillman reaction of aldehydes with enones or acrylates.

**Figure 14:**
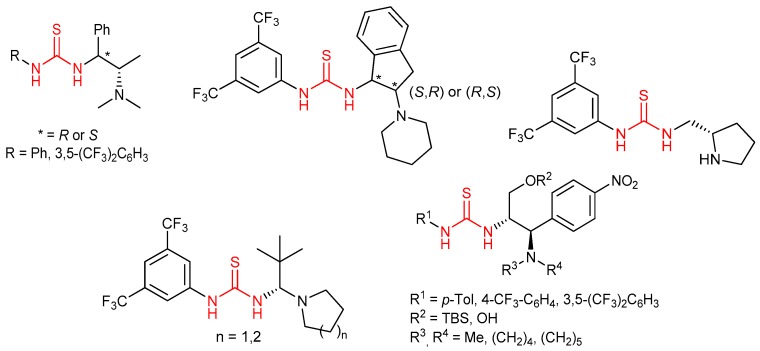
Chiral thioureas based on various diamines [187,188,189,190,191,192,193].

Other chiral thioureas bearing additional amino functionalities have been described as well (Figure 14). Ephedrine-based thioureas were obtained in Bolm’s group and tested in Michael additions [187]. Indane scaffold introduced into a structure of a catalyst resulted in good yields and stereochemical outcome of cascade Michael-oxa-Michael-tautomerization process [188]. A bifunctional pyrrolidine-thiourea was applied by Tang and co-workers to the diastereo- and enantioselective Michael additions of cyclohexanone to alkyl and aryl nitroolefins [189]. In a study by Enders and co-workers, tertiary amines (piperidine and pyrrolidine derivatives) were found the best option for domino Michael–Mannich cycloadditions [190]. Chen’s group prepared a series of chiral catalysts, including thioureas, using a chloramphenicol base skeleton, and used them in asymmetric transformations [191,192,193].

**Scheme 29:**
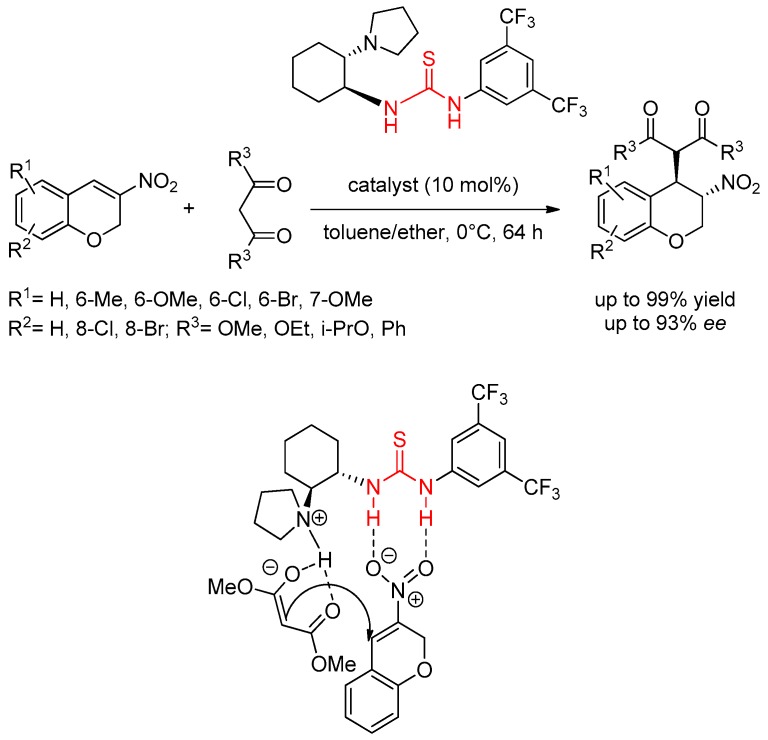
Asymmetric Michael addition and a proposed mechanism of formation of nitrochroman [197].

Chromans can be easily substituted using the Michael addition approach. Various chiral organocatalysts have been used in asymmetric reactions of nitroalkenes with malonates [194,195,196]. Thiourea-based ones bearing diamine functions were tested in the Michael addition by Yan and co-workers (Scheme 29) [197]. Tang’s group prepared chroman derivatives by addition of malonate to the appropriate nitroolefins in high yield and enantiomeric excess [189].

**Scheme 30:**
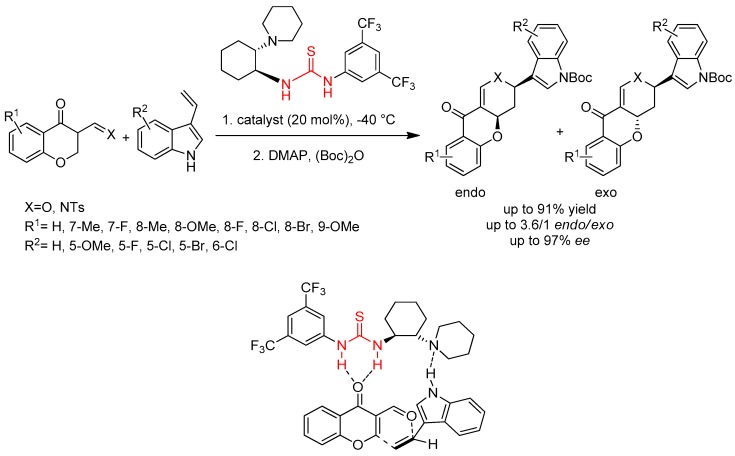
Asymmetric DA reaction and proposed interactions in a transition state [157].

Zhu and co-workers proposed a versatile method using vinylindols as dienophiles in an inverse electron demand Diels-Alder reaction (Scheme 30) [157,198]. This appeared an efficient method of preparation of new biochemically active flavonoids containing additional privileged structures such as pyran and indole [199].

**Scheme 31:**
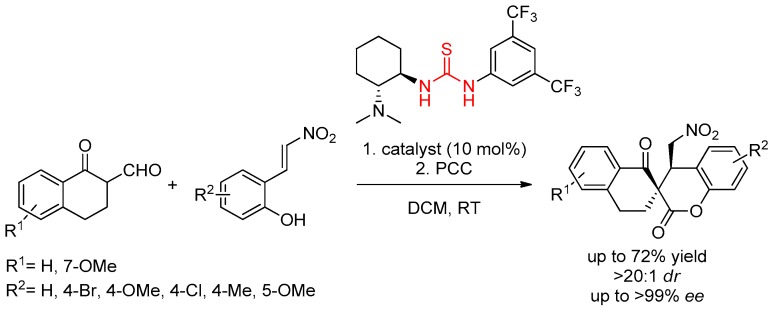

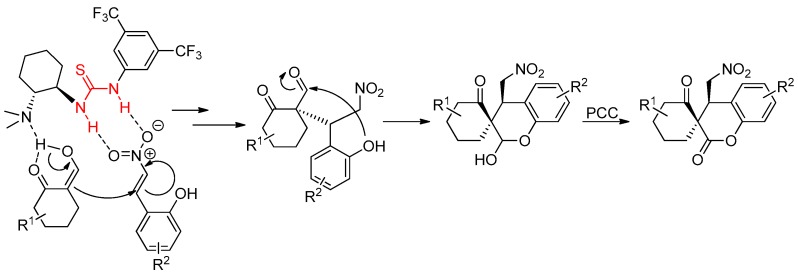
Asymmetric Michael-acetylation-cascade reaction and proposed reaction mechanism of spiro-center formation [212].

Over recent years, asymmetric cascade reactions [200,201,202,203,204,205] have been developed into a powerful tool in asymmetric synthesis of such compounds; stereogenic quaternary all-carbon centers are especially challenging [206]. Spiro-substituted structures can be found in biologically active natural and synthetic compounds [207,208]. The study on the asymmetric synthesis of spiro centers has been recently intensified [209,210], and various asymmetric routes to chromans bearing this structural feature have been found [208,211]. Thiourea-based multivalent organocatalysts offer a great opportunity for preparation of substituted spiro-chroman structures, as exemplified by Michael-acetylation-cascade reaction of 2-oxocyclohexanecarbaldehyde derivatives (Scheme 31) [212].

**Scheme 32:**
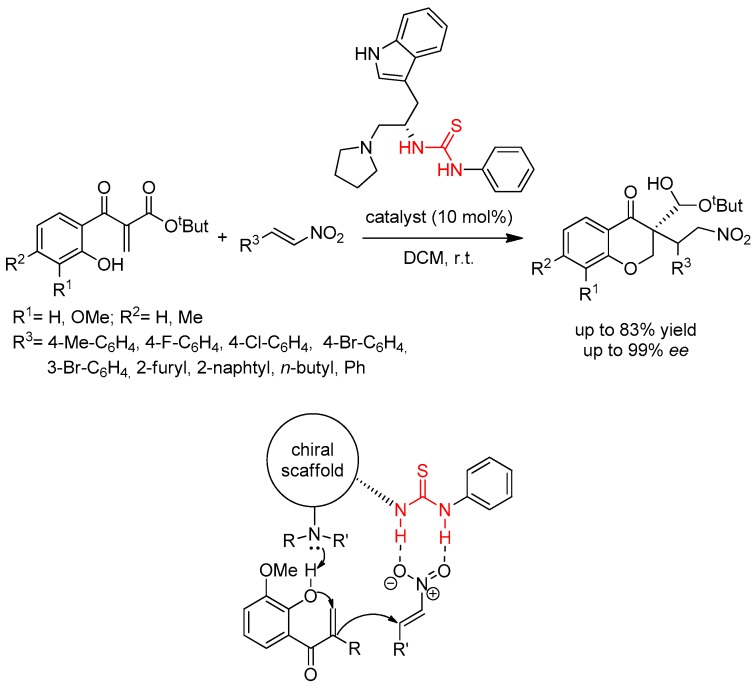
Formation of all-carbon quarternary centers catalyzed by a chiral thiourea and its postulated interactions with the reactant [213].

A non-spiro stereogenic quaternary all-carbon center was formed in an oxa-Michael addition followed by an intramolecular Michael addition as reported by Lu and co-workers (Scheme 32) [213]. 

### 3.2. Thioureas Containing Cinchona Alkaloids

**Figure 15:**
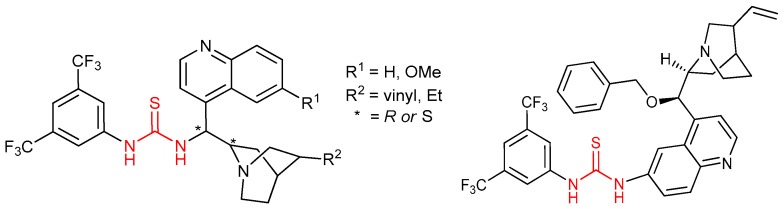
Thioureas derived from *Cinchona* alkaloids [216,217,250].

*Cinchona* alkaloids, a privileged motif of various structures useful in asymmetric synthesis [214,215], were also combined with thioureas, typically by conversion of hydroxyl group at C_9_ position into amine and its reaction with an appropriate isothiocyanate [17]. Such derivatives, introduced by the groups of Connon and Soós (Figure 15), were first applied in enantioselective addition of malonates to nitroalkenes [216] and nitroalkanes to chalcones [217]. Later on, these bifunctional organocatalysts were found efficient in a variety of asymmetric transformations, including Michael and combined Michael–Henry reaction [218,219,220,221,222,223,224,225,226,227,228,229,230,231,232,233,234,235,236,237,238], sulfa-Michael and retro-sulfa-Michael reaction [239,240], aldol reaction [241], Mannich reaction [242], Strecker reaction [243,244], hydrophosphonylation [245,246], decarboxylative protonation [247], fluorination of ketoesters [248], arylation of cyclic ketoamides with quinone monoamine [249] and others.

**Figure 16:**
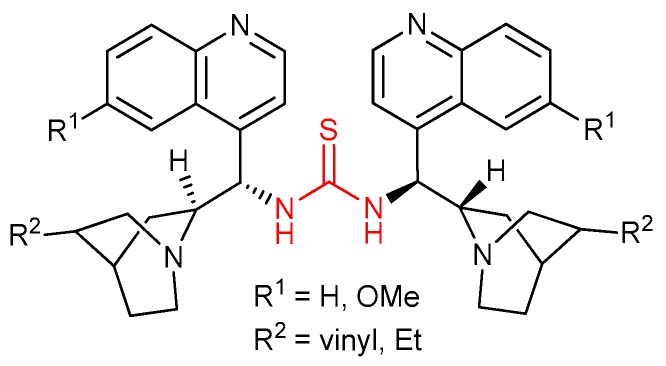
Bis-alkaloid thioureas prepared by Song’ group [251].

Thiourea was also introduced in place of methoxy group of quinine by Hiemstra and co-workers (Figure 16), and the resulting catalysts used in enantioselective Henry reaction [250], though they were less efficient in other asymmetric transformations [221,243,244,245]. Bis-alkaloid thioureas were prepared by Song and co-workers and were shown to exhibit high enantioselectivity in a dynamic kinetic resolution of racemic azlactone derived from valine (Figure 16) [251].

One can choose among stereoisomers (quinine/quinidine, cinchonidine/cinchonine and their epimers), and their modified derivatives, which often allows obtaining both optical antipodes of the product of the catalytical reaction [228,232,238,252]. However, other bisfunctional catalysts seem to offer more possibilities of fine tuning of catalytic properties. Certain problems are connected with limited thermal stability [217] and dimerization of alkaloid-thiourea conjugates in solution through intermolecular hydrogen bonds which can limit their activity [253,254].

**Scheme 33:**
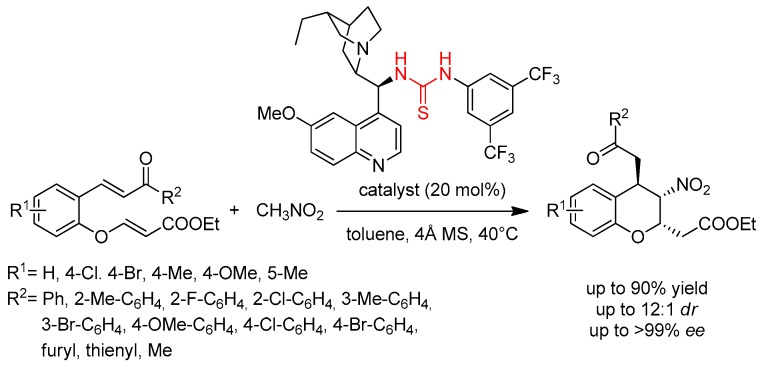

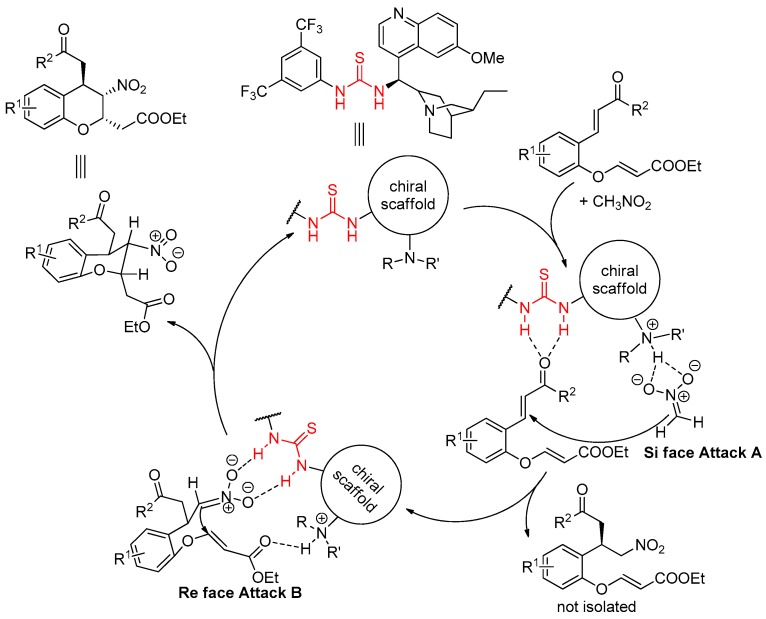
Asymmetric double Michael reaction catalyzed by *Cinchona* alkaloid-derived thiourea and a proposed reaction mechanism [255].

Gu and co-workers prepared a series of chroman derivatives in a double Michael reaction of commercially available starting materials, chalcone enolates and nitromethane, using *Cinchona*-alkaloid thioureas as catalysts (Scheme 33) [255]. Three stereogenic centers were formed with high stereoselectivity. In a proposed mechanism, both *Si* (not observed) and *Re* face approaches were included, which would result in a completely diverse hydrogen bonding interactions in a transient state.

**Scheme 34:**
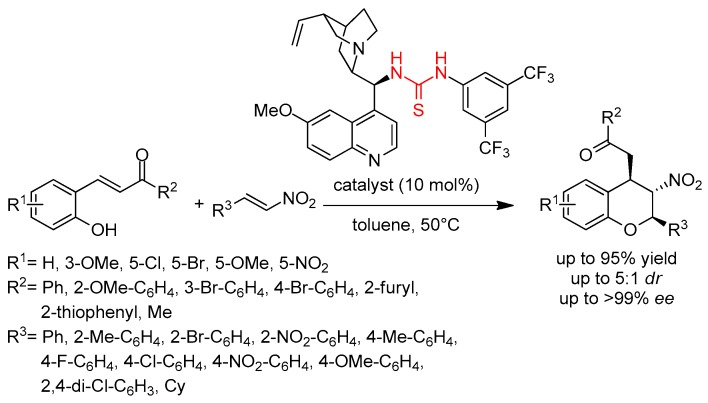
Synthesis of chiral chroman derivatives catalyzed by a thiourea obtained from *Cinchona* alkaloid [256].

*Cinchona* alkaloid-based organocatalysts were also applied by Singh and co-workers in their synthesis of chiral chroman derivatives from chalcones and α,β-unsaturated nitroalkenes (Scheme 34) [256]. Reasonable yields were accompanied by a modest diastereoselectivity and high enantioselectivity, and the reaction was completed in 8–48 h.

### 3.3. Thioureas Derived from Amino Acids and Peptides

**Figure 17:**
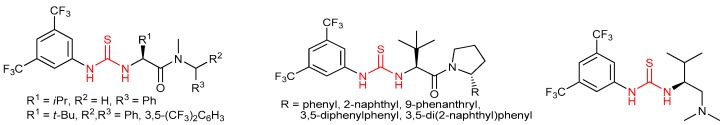
Examples of chiral thioureas derived from amino acids [263,264,265,266,267,268,269].

As already demonstrated by Jacobsen’s catalyst (described in part 3.2.) [123], naturally occurring amino acids and their derivatives can serve as chirality source in the construction of chiral thioureas [62,257,258,259,260]. Jacobsen’s group described the application of amide-thiourea catalyst obtained in three steps from valine and *N*-methylbenzylamine in Pictet-Spengler reaction [261,262], and *tert*-leucine-derived amides in iso-Pictet-Spengler reaction (both reactions were co-catalyzed with benzoic acid; Figure 17) [263]. Thioureas bearing *t*-leucine arylpyrrolidino amide component catalyzed the stereoselective indole addition to γ-pyrone derivatives [264]. *t*-Leucine-derived thioureas were also efficient in the enantioselective dearomatization of isoquinolines [265], and the synthesis of furan derivatives with a trifluoromethyl at a stereogenic center [266]. Valine derivative prepared by Pedrosa and co-workers was applied in nitro-Michael additions; a supported catalyst was also prepared [267,268,269]. Fullerene was also introduced into chiral thioureas derived from valine, phenylalanine and *tert*-leucine, and the resulting hybrids appeared efficient, recyclable catalysts for stereoselective nitro-Michael reaction [270].

**Figure 18:**
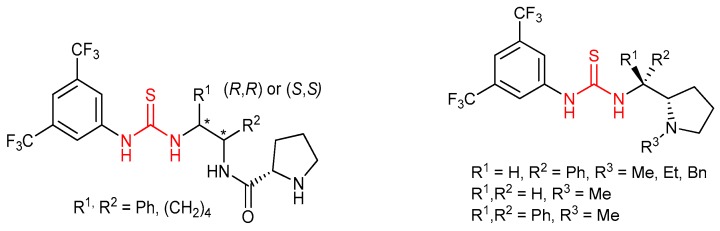
Chiral thioureas obtained from l-proline [271,272,273].

Thioureas derived from l-proline were reported by various groups (Figure 18) [271,272,273]. These derivatives were applied in asymmetric direct aldol reaction of cyclohexanone and aldehydes or enantioselective Michael addition of 1,3-diones to nitroolefins, both proceeding in high yields and with excellent stereoselectivity [271,273].

**Figure 19:**
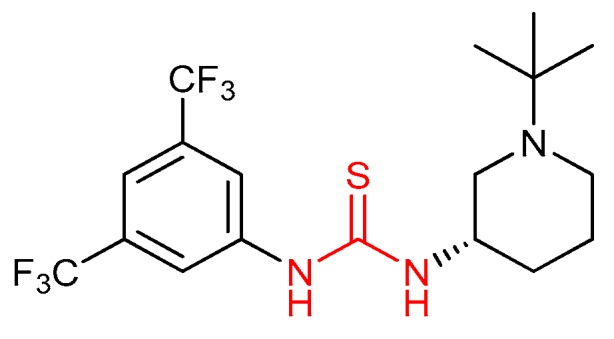
An example of chiral thiourea prepared in Bolm’s group [274].

A series of thioureas was obtained by Bolm and co-workers starting from l-aspartic acid and l-glutamic acid which were converted into cyclic amines and reacted with organic isothiocyanates (Figure 19) [274]. The resulting compounds provided excellent yields and stereoselectivities of Michael additions.

**Figure 20:**
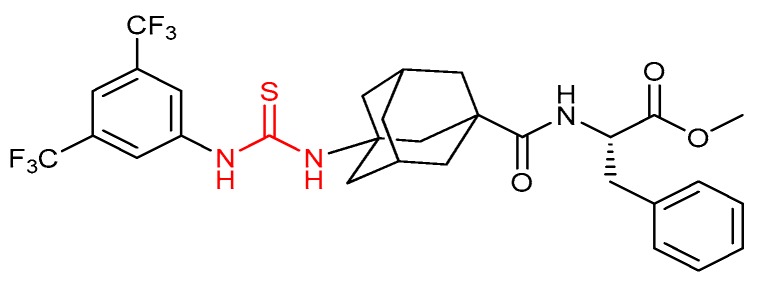
A peptide-like chiral thiourea [276].

In 2007, Lattanzi prepared thioureas from commercially available enantiopure aminoalcohols and used them in stereoselective Morita–Baylis–Hillman reaction of cyclohexanone and aldehydes [275]. Various peptidomimetics containing thiourea fragment were prepared as well [39,79,90]. Peptidic derivatives containing adamantyl moiety were found efficient catalysts of Morita–Baylis–Hillman reactions (Figure 20) [276].

**Scheme 35:**
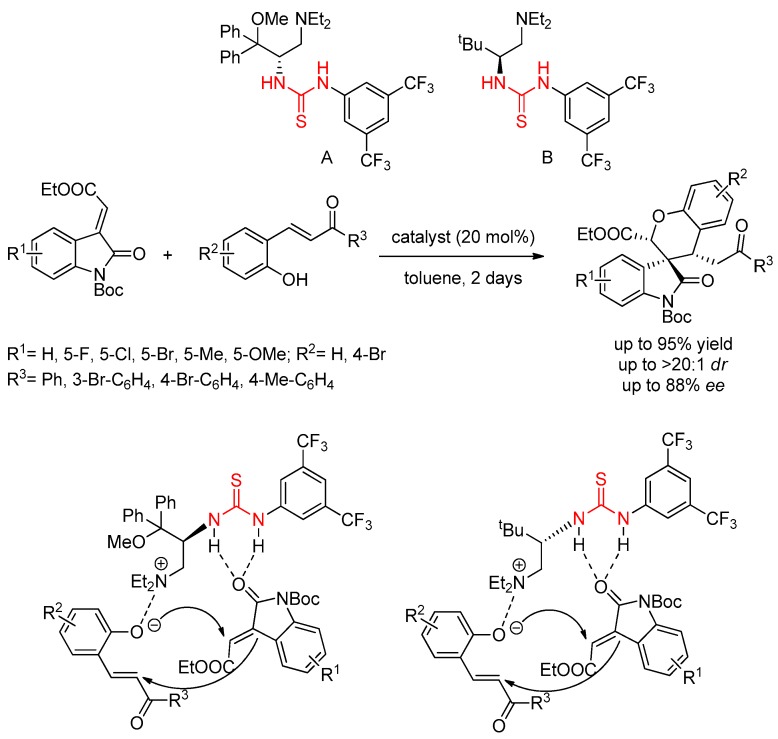
Asymmetric tandem Michael–Michael reaction reported by Zhao et al. and its proposed mechanism [277].

Amino acid-derived thiourea organocatalysts were used by Zhao’s group in a tandem thia/oxa Michael-Michael reaction yielding optically pure chromans containing the pharmaceutically important oxindole scaffold (Scheme 35) [277]. Spiro products were isolated in high yields and good up to very high enantiomeric excess.

**Scheme 36:**
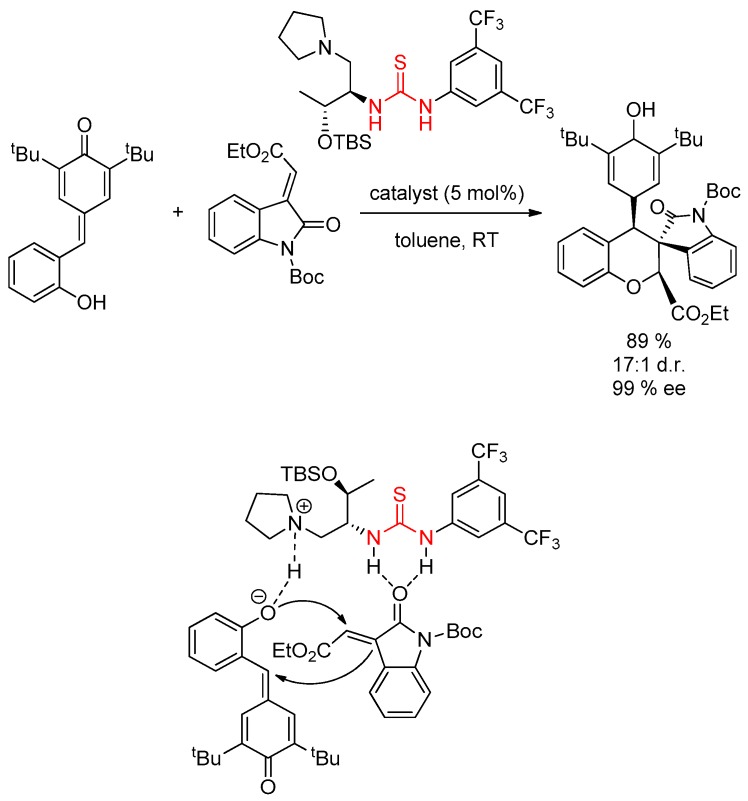
Asymmetric domino oxa-Michael-1,6-addition and the proposed interactions in the transient state [278].

Excellent yield, diastereomeric and enantiomeric excess were reported in a domino reaction of oxa-Michael and 1,6-addition by Enders et al. (Scheme 36) [278]. As privileged substrates *ortho-*hydroxyphenyl derivatives were used [279], introducing a nucleophilic group *para-*quinone methides that became suitable donor-Michael acceptors [280,281,282,283,284,285,286,287].

### 3.4. Carbohydrate-Based Chiral Thioureas

Chiral thioureas can bear enantiopure carbohydrates. Quite frequently, they are combined with other functionalities. For example, Liu et al. prepared five novel d-mannitol-derived thiourea organocatalysts containing *Cinchona* alkaloids as well and used them for asymmetric Henry reaction [40].

**Figure 21:**
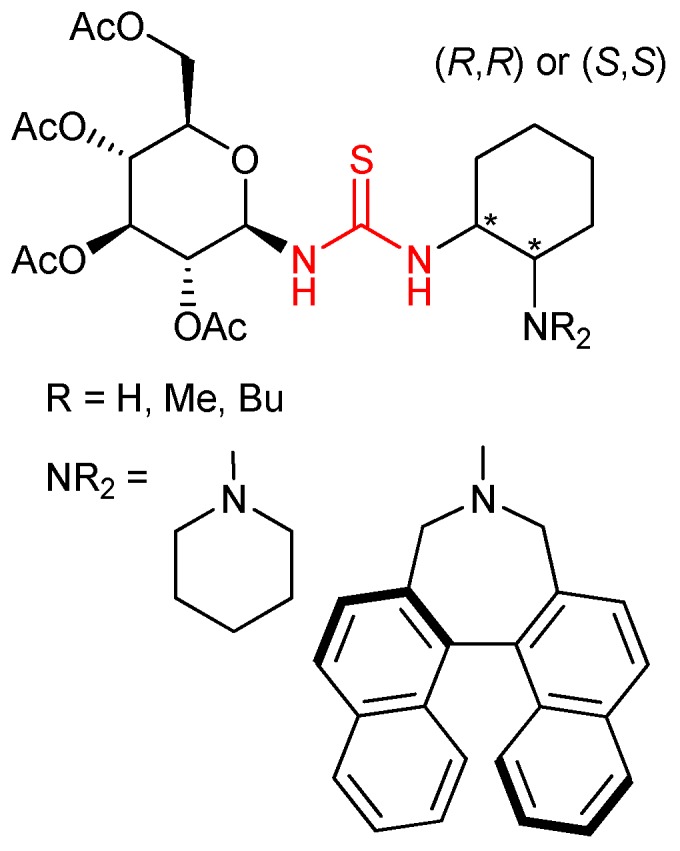
Carbohydrate-derived chiral thiourea [288].

Ma and co-workers introduced bifunctional organocatalysts containing multiple stereogenic centers in both primary amine derived from DACH and various carbohydrate moieties (Figure 21) [288]. With these promoters, Michael addition of ketones to nitroalkenes proceeded with high yields and stereoselectivities. Similar catalysts (also containing 1,2-phenylenediamine) were applied in other Michael additions [194,289], Mannich reaction [290,291], and conjugate addition/dearomative fluorination [292,293]. Thioureas containing a glycosyl scaffold were also applied in aza-Henry reaction between *N*-Boc imines and nitromethane [294].

**Scheme 37:**
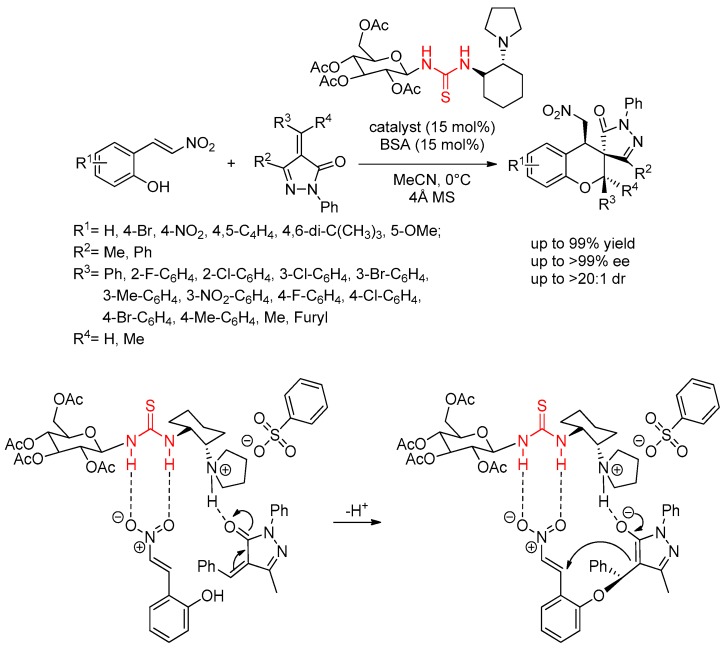
Asymmetric synthesis of various spiro-compounds with mechanism proposed [295].

Asymmetric tandem Michael–Michael additions of ketones and nitroalkenes were optimized by Miao and co-workers (Scheme 37) [295]. A carbohydrate-based chiral thiourea organocatalyst used together with benzenesulfonic acid (BSA) afforded high yields, excellent enantiomeric excess and high diastereoselectivity. The resulting spiro-compounds are not only interesting due to possible biological activities [296], but also as enantiopure multi-functionalized pyrazole derivatives for further synthesis [211,212,297,298,299].

### 3.5. Chiral Phosphine-Bearing Thioureas

**Figure 22:**
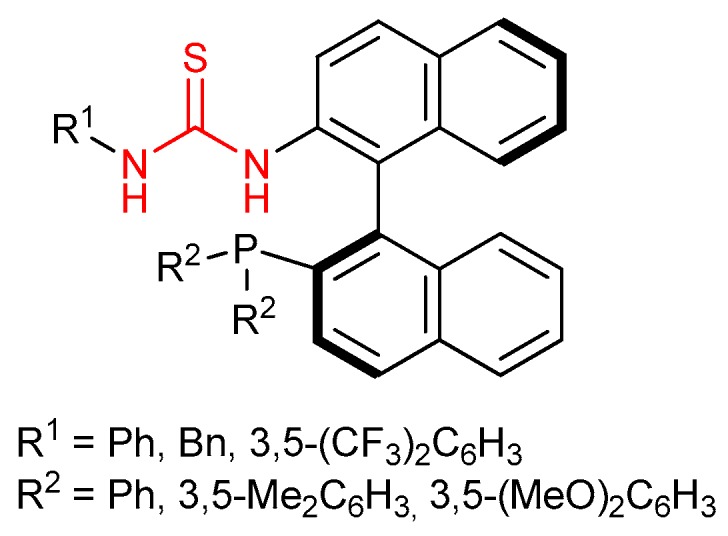
Axially chiral phosphine-containing thiourea [300].

Bifunctional catalysts containing thiourea moiety (hydrogen bond donor, Brønsted acid) and nucleophilic/basic phosphine functionality, both attached to a chiral skeleton, have already found a variety of applications in stereoselective reactions [113]. The first report on their synthesis was published by Shi and Shi in 2007 who prepared three binaphthyl-based derivatives (Figure 22) [300]. A *N-*phenyl-substituted catalyst was most efficient in aza-Morita-Baylis-Hillman reaction of *N*-sulfonated imines and vinyl ketones or acrolein: (*S*)-products were formed in moderate to high yield and stereoselectivities. Shi and co-workers used modified thioureas belonging to the same class in the reaction of MBH adducts with oxazolones [301]. 

**Figure 23:**
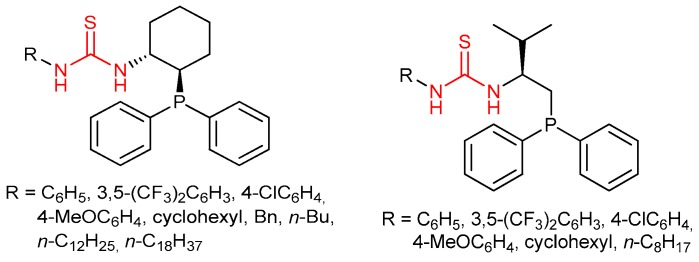
Chiral thioureas with diphenylphosphine moiety [302,303].

An active catalyst bearing a diphenylphosphine moiety was introduced by Wu and co-workers and tested for the Morita–Baylis–Hillman reaction of aromatic aldehydes with methyl vinyl ketone and acrylates (Figure 23) [302,303]. Slightly higher (and opposite) enantioselectivities were noted for valine-derived phosphinothiourea, however, the reaction required more time [304].

**Figure 24:**
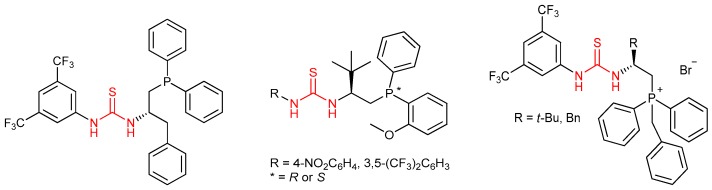
Examples of chiral thioureas bearing phosphine functionalities[305,307] and a phosphonium salt [308].

Phosphinothiourea, described by Mita and Jacobsen, catalyzed enantioselective opening of aziridines with hydrogen chloride to yield β-chloroamine derivatives (Figure 24) [305]. Chiral thiourea bearing a phosphine moiety was also efficient in 1,6-conjugate addition of *para*-quinone methides with dicyanoolefins [306]. An interesting example of thioureas bearing a stereogenic phosphorus atom, prepared by a stereoselective reduction of the corresponding aminophosphine oxides and their reaction with isothiocyanates, was described by Su and Taylor (Figure 24) [307]. Epimeric catalysts exhibited different activity and stereoselectivity in Morita-Baylis-Hillman reactions of methyl acrylate and aromatic aldehydes.

In a 2019 paper, amino acid-derived thiourea-phosphonium salts were explored as phase-transfer catalysts for asymmetric Mannich reaction (Figure 24) [308].

**Figure 25:**
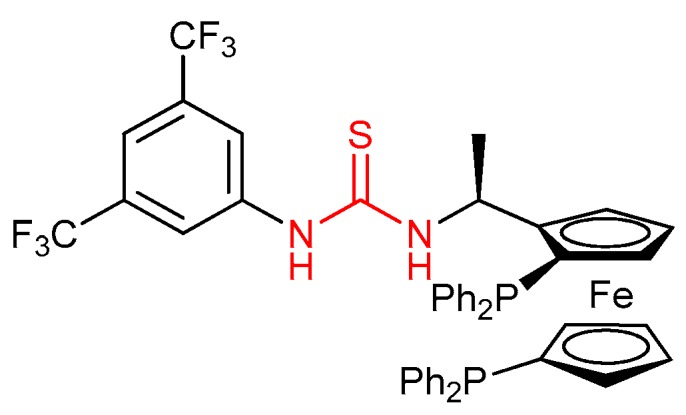
ZhaoPhos, a chiral bis-phosphine thiourea organocatalyst [309,310].

A novel chiral ferrocenyl bis-phosphine thiourea was introduced by Zhang and co-workers who described its use in Rh-catalyzed hydrogenation of nitroalkenes with high yields and enantioselectivities, even at low catalyst loading (Figure 25) [309,310]. This ligand, named ZhaoPhos, easily prepared from Ugi’s amine, showed high efficiency in various hydrogen bond-assisted catalytic hydrogenations with rhodium and iridium complexes [311,312,313,314,315,316,317,318]. The system exemplified the idea of synergistic activation via cooperating transition metal-catalyst and organocatalyst joined in one bifunctional structure [319].

Other chiral phosphorus derivatives of thioureas are also worth mentioning. Two phosphorylamide derivatives were prepared by Juaristi and co-workers and utilized in stereoselective Michael addition of cyclohexanone to nitrostyrenes and chalcones [42]. Chiral thioureas containing an aminophosphonate moiety were investigated in context of their antiviral activity as well [320]. 

**Scheme 38:**
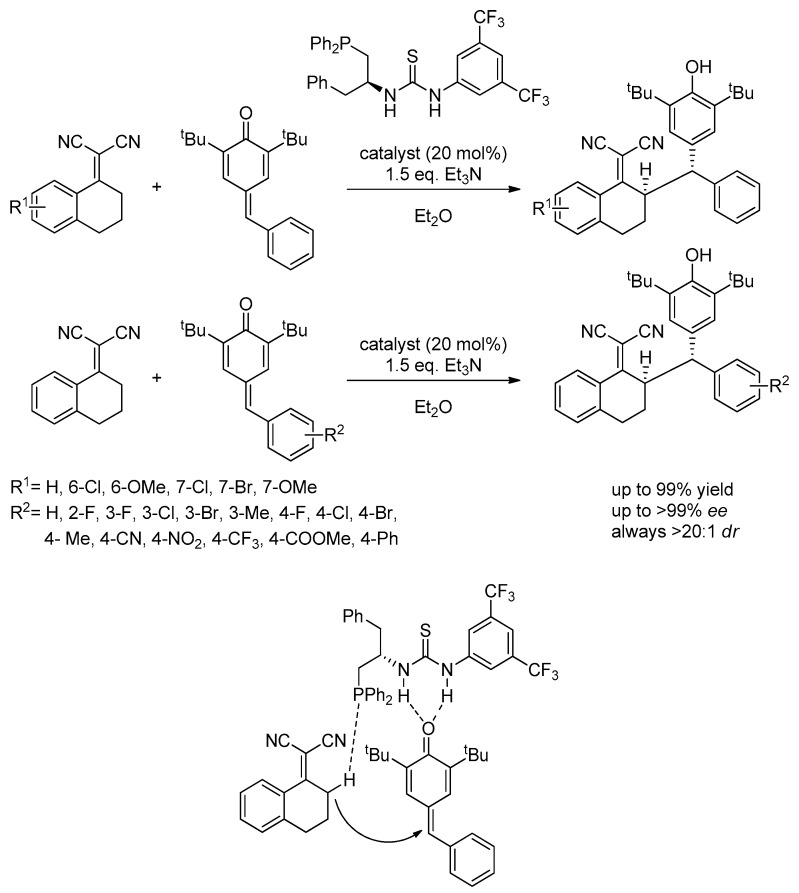
Asymmetric reaction of *para*-quinone methides catalyzed by chiral phosphine thiourea catalysts and a proposed transient state [306].

In a chiral phosphine thiourea-mediated reaction of *para-*quinone methides with dicyanoolefins described by Yao et al. excellent diastereomeric excess in combination with good yield and enantioselectivity were observed (Scheme 38) [306]. 

### 3.6. Thioureas and Bis-Thioureas with Axial, Planar or Helical Chirality

Chirality of majority enantiopure thioureas used in asymmetric organocatalysis origins from the presence of stereogenic center(s). However, one cannot neglect a group of derivatives which are characterized by the presence of other stereogenic elements. Some notable representatives include efficient organocatalysts and exciting examples of molecular motors from Feringa’s laboratory.

**Figure 26:**
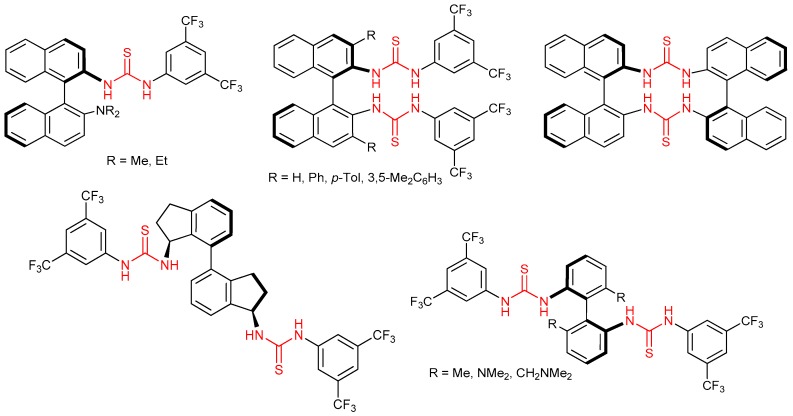
Examples of thioureas and bis-thioureas with axial chirality [28,29,323,324,325,326,327,328].

Most compounds belonging to this class exhibit axial chirality/helicity connected with the presence of biaryl fragment characterized by a restricted rotation around the C_aryl_–C_aryl_ bond. First examples come from 2005, when, looking for optimal organocatalyst for the asymmetric Morita-Baylis-Hillman reaction of cyclohexanone with aldehydes, Wang and co-workers decided to combine thiourea moiety with binaphthylamine (Figure 26) [28]. An axially chiral binaphthyl bis-thiourea was prepared by Connon and co-workers and proved useful in promotion of asymmetric Friedel–Crafts type addition of indole derivatives to nitroalkenes (Figure 26) [29]. Modified catalysts belonging to this class obtained in Shi’s group were also efficient in the asymmetric Henry [30] and Morita-Baylis-Hillman reactions [300,301,321]. A multifunctional organocatalyst containing quinine, thiourea and binaphthylamine moieties, capable of stereoselective formation of three stereocenters in a domino Michael-aldol reaction was constructed by Barbas III and co-workers [322]. Novel bis-thioureas prepared by Rampalakos and Wulff from commercially available enantiomerically pure 1,1′-binaphthyl-2,2′-diamine were tested in aza-Henry reactions (Figure 26) [323]. A series of binaphthyl-derived thiourea catalysts were applied by Kim and co-workers in asymmetric Mannich-type reactions of fluorinated ketoesters [324]. For the asymmetric Henry reaction, bis-thioureas were prepared connected with 4,4′-bisindanyl [325], and substituted biphenyl- and bianthryl-based linkers (Figure 26) [326,327,328]. High yields and stereoselectivities were observed for certain derivatives.

**Figure 27:**
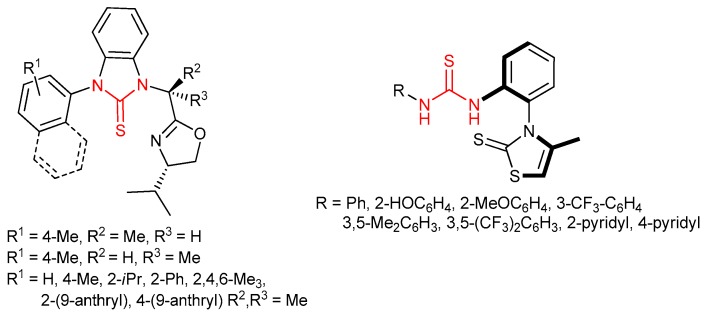
Thioureas with axial chirality resulting from a restricted rotation [82,329].

A combined point chirality and axial chirality resulting from the restricted rotation was observed for thiourea-oxazoline ligands used for palladium-catalyzed asymmetric reactions (Figure 27) [82,88]. A series of enantiopure atropisomeric thioxothiazol-substituted derivatives prepared by Roussel et al. were tested as enantioselective anion receptors (Figure 27) [329].

**Figure 28:**
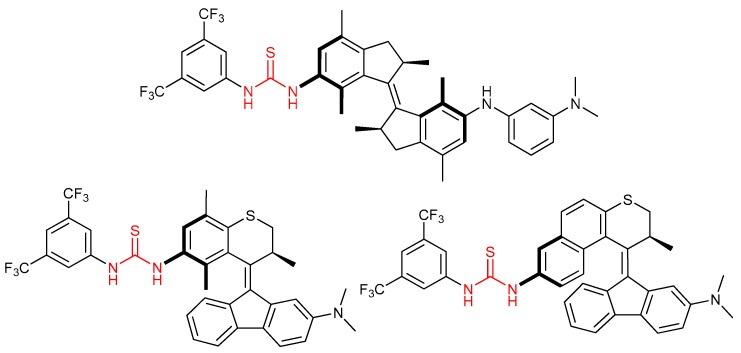
Molecular motors bearing thiourea moiety [330,331].

Bisfunctional, photoswitchable, dual stereoselective catalysts based on the idea of unidirectional molecular motors were designed by Feringa and co-workers (Figure 28) [330,331]. The cooperation of thiourea and tertiary amine in *cis* states was found a key factor for stereoselectivity of Henry reaction of nitromethane and fluorinated ketones and Michael reaction of bromonitrostyrene and pentanedione.

**Figure 29:**
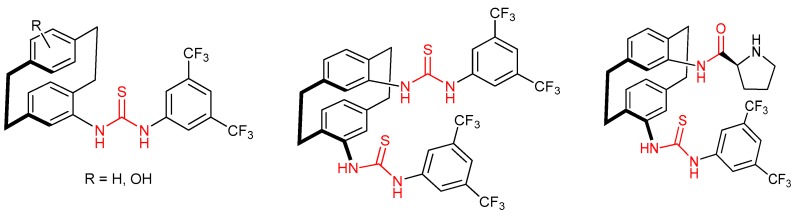
Examples of thioureas with planar chirality [31,32,33].

Enantiomerically pure planar-chiral thiourea derivatives based on [2.2]cyclophane were first prepared by Paradies and co-workers and showed rather limited efficiency in stereoselective Friedel–Crafts alkylation of indole and transfer hydrogenation of nitroolefin (Figure 29) [31]. However, the performance of cyclophane bis-thioureas in asymmetric Henry reaction was much improved and also led to high induction of chirality (Figure 29) [32]. Other modifications of the system (the introduction of amino group) were found useful for the catalysis of aldol reaction of isatines and ketones (Figure 29) [33].

Thioureas were also incorporated to optically active helical polymers. Polymerized enantiopure *N*-propargylthioureas derived from 1-phenylethylamine formed helices in low polarity solvents and showed affinity to iron(III) ions [332]. Polyacetylenes bearing pendant thiourea groups were used as chiral catalysts for the asymmetric Michael addition of diethyl malonate to trans-β-nitrostyrene [333].

### 3.7. Thioureas Containing Other Functional Groups

**Figure 30:**
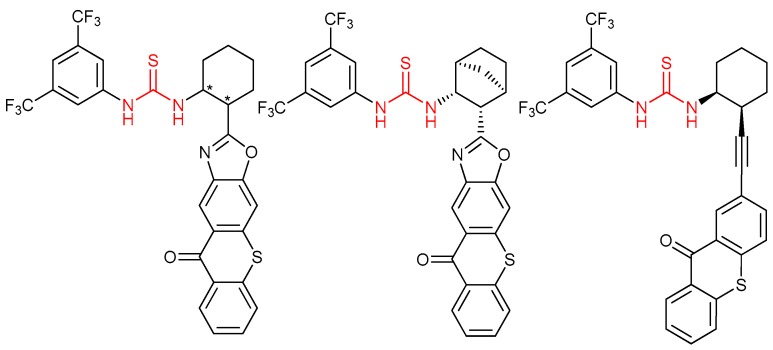
Chiral thioureas bearing thioxanthone fragment [334].

A variety of thiourea organocatalysts have been prepared containing additional functionalities to introduce chirality or/and to actively participate in the catalytic reaction. For example, photochemically active thioxanthone was attached to thiourea moiety with various chiral linkers and the resulting catalysts were employed in the photocyclization of 2-aryloxycyclohex-2-enones (Figure 30) [334].

**Figure 31:**
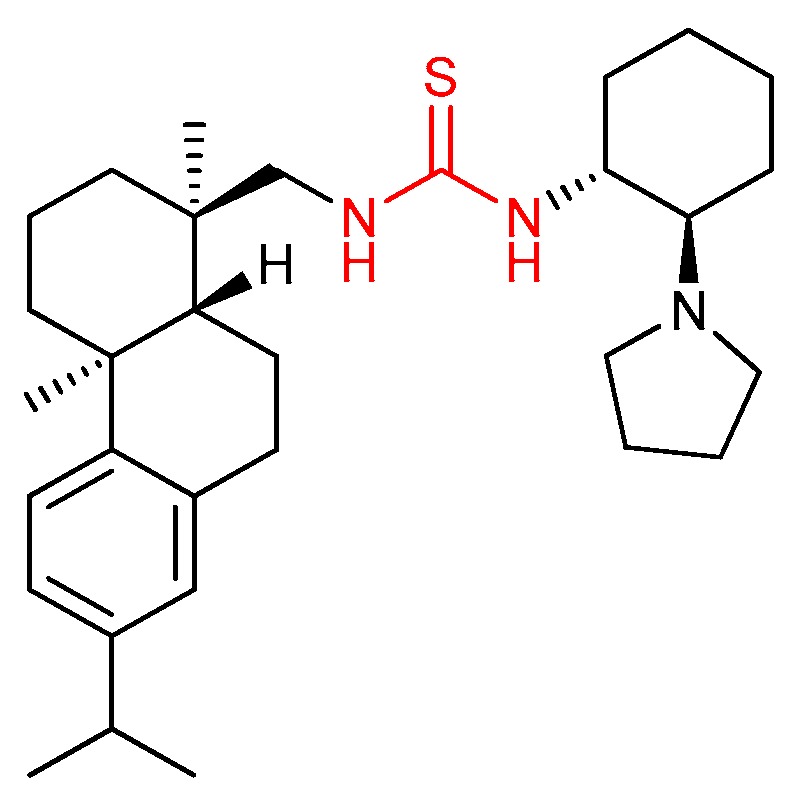
Rosin-derived thiourea [51].

Wang and co-workers reported a preparation of bifunctional rosin-derived catalysts (prepared from chiral dehydroabietic amine) and used it in a doubly stereocontrolled addition reactions, including the stereoselective synthesis of chiral cyclic thioureas (Figure 31) [51,171,335,336,337,338,339,340,341]. Wang’s group modified the original structure of their catalyst with *Cinchona* alkaloids as well to provide a double stereocontrol [342,343,344]. Rosin-derived thioureas were also used by Reddy and co-workers in asymmetric Michael-hemiketalization of allomaltol [345]. 

**Figure 32:**
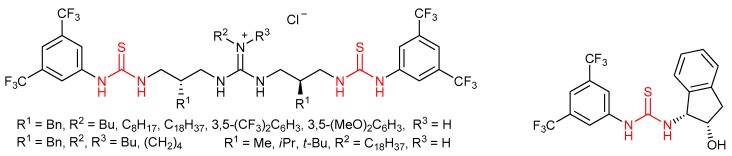
Chiral thioureas with guanidine moiety [346] and indanol derivative [347].

A series of chiral bis-thioureas bearing a central guanidine motif were developed by Nagasawa’s group and applied as catalysts of Henry reaction of nitromethane and various aldehydes (Figure 32) [346]. Ricci’s group developed an indanol chiral thiourea organocatalyst for enantioselective Friedel-Crafts alkylation of indoles with nitroalkenes (Figure 32) [347].

**Figure 33:**
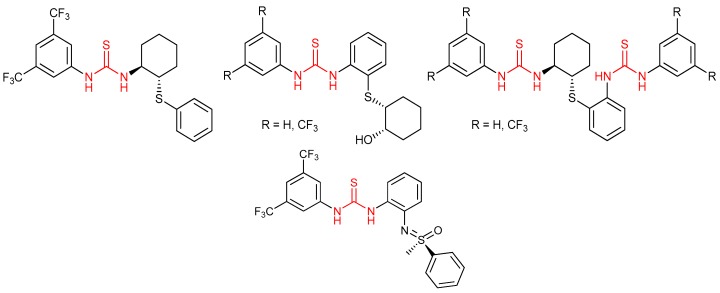
Chiral thioureas substituted with thioether groups [348] and sulfoximine [41].

Thioureas can be equipped with additional sulfur functionalities. In our laboratory, chiral amino- and diaminosulfides obtained from cyclohexane-1,2-diol were converted into mono- and bisthioureas which were tested in Morita-Baylis-Hillman reaction (Figure 33) [348]. Bolm and co-workers designed the synthesis of a set of chiral sulfoximine-based thioureas (Figure 33) [41]. Interestingly, the optimum results of asymmetric Biginelli reaction were obtained for the derivative bearing only sulfur as a stereogenic center separated from thiourea moiety.

Krasonvskaya et al. described a preparation of a thiourea-modified doxorubicin as a pH-sensitive prodrug capable of releasing cytotoxic component as well as anticonvulsant albutoin in a weakly acidic medium [349]. Thioureas containing a chiral isosteviol moiety [350,351,352], terpenes [77], camphor [353] or steroid scaffold [354] have been also reported.

## 4. Biological Activity of Chiral Thioureas 

Thioureas represent an important class of compounds that attracted a lot of attention due to their bioactivity, e.g., in medicinal chemistry, pharmaceutical industry, and agriculture. Derivatives were found to be efficient antiviral [72], antifungal [355], antimicrobial [356] or anticancer agents [357]. The ability of preparing thioureas containing various scaffolds allows their use as potential inhibitors against numerous molecular targets. Consequently, these compounds have become an interesting material for further investigations. In this part of the review, selected examples of chiral thiocarbamides used in biomedical studies will be presented.

### 4.1. Antiviral Thioureas

Aminophosphonates are structural analogues of amino acids. Likewise the aminophosphonic acids, they are able to inhibit enzymes involved in amino acids metabolism, and thus may generate various physiological responses, e.g., neuromodulatory activity [358]. Cucumber mosaic virus (CMV) and tobacco mosaic virus (TMV) are plant virus diseases which have become a serious problem in agriculture in recent years [359]. The commercially available product Ningnanmycin is commonly used against CMV and TMV, though with serious limitations resulting from its light and moisture sensitivity. Thus, the design and preparation of novel antiviral agents constitutes a significant challenge.

**Figure 34:**
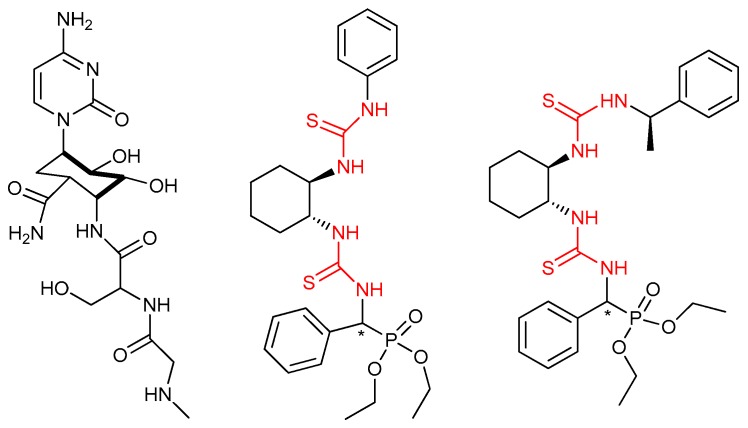
Ningnanmycin (left) and the most active bis-thiourea derivatives bearing α-aminophosphonate functional group [320,359].

In 2009, Chen et al. reported the novel anti-TMV chiral thioureas and bis-thioureas bearing α-aminophosphonate moiety [320]. The evaluation of a tested series revealed that two derivatives are active against CMV and TMV comparable to Ningnanmycin (Figure 34). It was also found that the absolute configuration of isothiocyanates, used to form corresponding mono- and bis-thioureas, considerably affected the antiviral activity of tested compounds. Generally, mono-thiourea (*S*)-enantiomers were more effective against TMV than corresponding (*R*)-enantiomers [320], while the results for bis-thioureas against CMV appeared to be reversed: derivatives obtained from (*R*)-enantiomer of isothiocyanate were more active against the virus [359].

**Figure 35:**
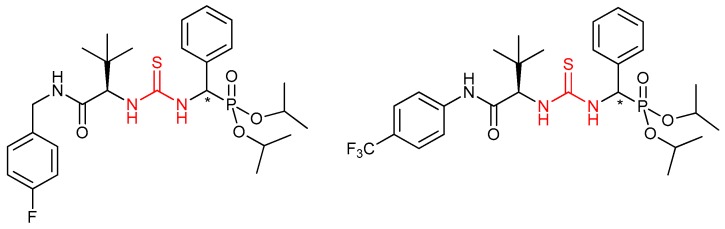
The most active fluorinated thiourea derivatives containing α-aminophosphonate moiety based on leucine [361].

Liu and co-workers prepared a series of chiral thioureas bearing *t*-leucine and α-aminophosphonate moieties and evaluated their anti-TMV activity [360]. Two compounds were identified as the most potent antiviral agents (Figure 35). It was shown that the derivatives containing electron withdrawing groups in *para* position of the aromatic ring revealed better activity. Stereoisomers with *l* configuration were more active than their *d* counterparts or the racemic form.

**Figure 36:**
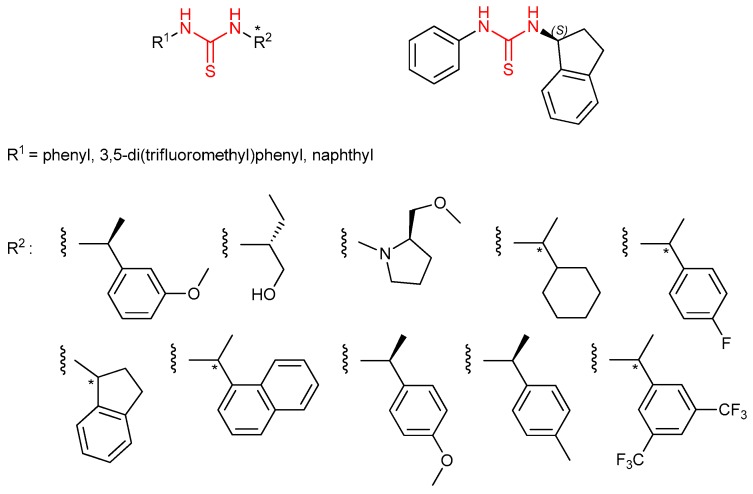
A set of new chiral thioureas (left) prepared by Yan et al.and the most active indane derivative (right) tested against TMV [361].

Yan et al. synthesized also a new collection of chiral thioureas and evaluated their activity against TMV [361]. Novel compounds were prepared in ionic liquid, an eco-friendly environment. Among them, an indane derivative (Figure 36) exhibited the best antiviral properties: in vivo protection, inactivation and curative effects against TMV with inhibitory effects of 57.0%, 96.4% and 55.0%, respectively, at 500 μg/mL. Moreover, it was more active against TMV than the reference compound Ningnanmycin.

Besides the agriculture, various thiourea derivatives are important for pharmaceutical industry. Several examples of structurally diverse derivatives that act on human viruses, e.g., human immunodeficiency virus (HIV) or human cytomegalovirus (HCMV) can be found in the literature [362,363].

After extensive studies of the pharmacological influence of novel 1,3-thiazepine-based urea and thiourea derivatives on animal central nervous system (CNS), Struga and co-workers published the results of their antiviral activity assays [364]. The compounds were found useful in antiviral therapy due to their specific structure: butterfly-like conformation formed by the hydrophilic center and two hydrophobic moieties. Such a structure is characteristic for non-nucleoside reverse transcriptase inhibitors (NNRTIs), used as anti-HIV agents [364,365].

Furthermore, the 1,3-thiazepine ring is an important system considering its biological activity. It is a part of the Omapatrilat structure, an antihypertensive drug currently in stage IV of clinical trials [366,367]. Additionally, seven-membered cyclic thiourea derivatives are used as a nitric oxide synthase inhibitors [366].

**Figure 37:**
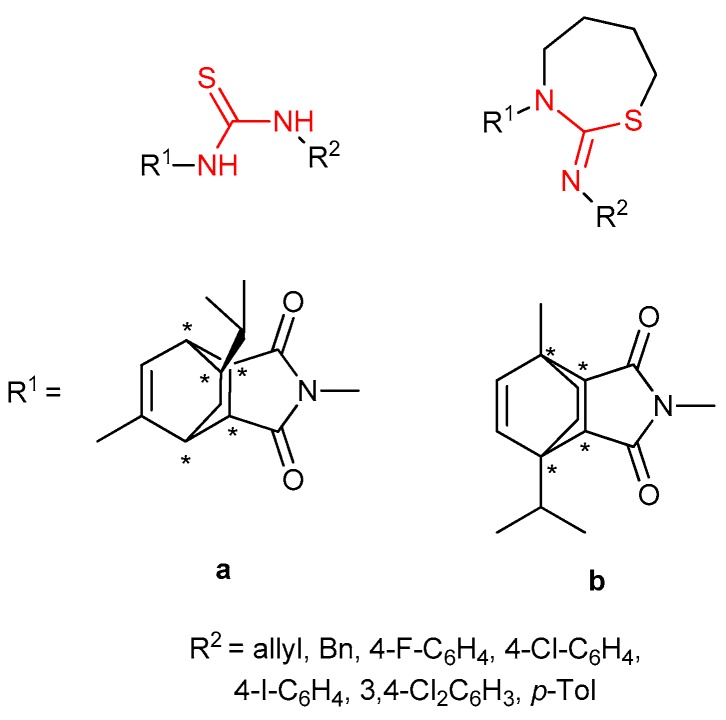
1,3-Thiazepine isothiourea derivatives [364].

The 1,3-thiazepine-based isothiourea derivatives were tested against diverse virus classes: Retrovirus (HIV-1), Hepadnavirus (HBV) and Flaviridae (YFFV and BVDV, both the single-stranded RNA^+^ viruses; Figure 37). In spite of the promising pharmacological action on animal CNS, only three compounds exhibited modest antiviral activity [364].

**Figure 38:**
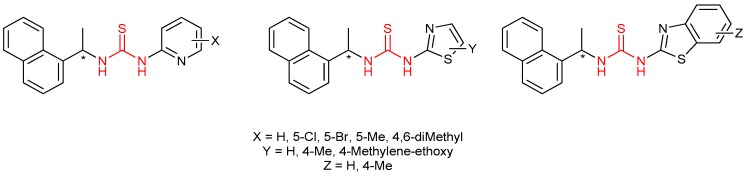
A series of chiral naphthyl thioureas tested as anti-HIV agents [368,369].

Venkatachalam et al. studied the impact of the stereochemistry of thioureas on their anti-HIV activity (Figure 38) [368]. Eleven chiral naphthyl thioureas were tested in vitro against recombinant reverse transcriptase (RT) [369]. Generally, the (*R*)-stereoisomers of all eleven compounds were more active than their enantiomers. Five of the most active compounds were further evaluated for their ability to inhibit HIV-1 replication in human peripheral blood mononuclear cells (PBMC). While the (*R*)-stereoisomers were active at nanomolar concentration, their enantiomers were again inactive. Furthermore, the most active compound was much more active against various NNI-resistant HIV-1 strains, than standard NNI drugs (nevirapine, delavirdine and trovirdine). Molecular modelling studies confirmed that the (*R*)-isomer fits to the target NNI binding pocket on HIV-RT much better than the (*S*)-enantiomer [368].

**Figure 39:**
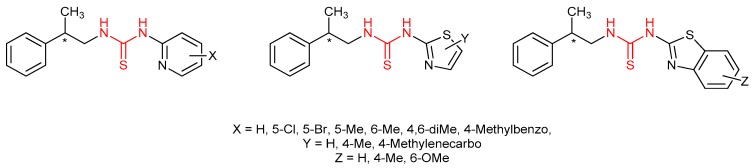
Chiral thioureas studied by Vankatachalam and co-workers as antiviral agents [372].

In the 1990s, Bell and co-workers reported phenethylthiazolylthiourea (PETT) compounds as potent anti-HIV agents; taking the structure-activity relationship into account various substituents in their structures were analyzed [370,371]. Later, Venkatachalam’s group undertook the studies of the influence of stereochemistry on the activity of this class of compounds [372]. A new series of chiral halopyridyl- and thiazolyl-substituted thioureas were synthesized (Figure 39). Molecular modelling suggested that for both groups (*R*) stereoisomers fit better to the target binding pocket of HIV reverse transcriptase than their (*S*) counterparts. The in vitro tests confirmed this result and exhibited also that the lead compounds were several orders of magnitude more potent than the standard NNRTI Nevirapine.

### 4.2. Anticancer Thioureas

Since cancer constitutes the second most common cause of death globally, the quest for the new anti-tumor agents remains a continuous interest of numerous scientific teams. It has been proven that various groups of thioureas exhibit antiproliferative activity [373,374,375]. Some compounds reveal dual biological effects, e.g. anti-tumor together with anti-oxidant or anti-inflammatory activity [376]. Depending on structure and content of other biologically active fragments they act on various types of cancer cells. Herein, we present chosen, promising results of antiproliferative studies.

**Figure 40:**
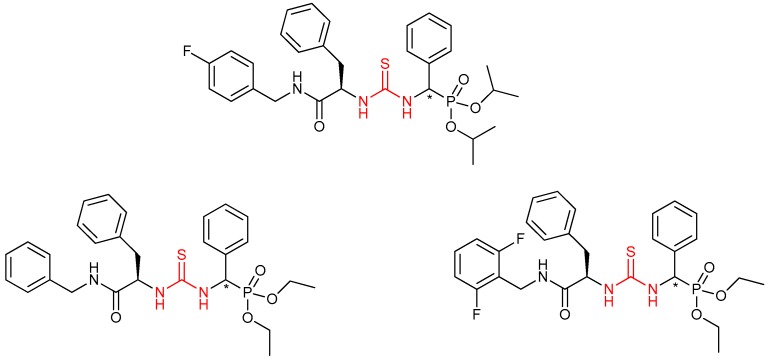
Potential lead compounds displaying antiproliferative activity in vitro [376,377,378].

Chiral thioureas containing α-aminophosphonate moiety, already mentioned as potential antiviral agents, were also used by Liu et al. as pseudo-peptides to treat cancer (Figure 40) [377]. Several derivatives revealed promising activity against human tumor cells PC3 (prostate cancer), Bcap37 (breast cancer) and BGC823 (stomach cancer). In a preliminary in vitro assay the three most potent compounds emerged with IC_50_ values from 4.7 to 17.2 µM against the PC3 cell line; two of them exhibited a better antiproliferative activity than the reference compound—a commercially available 6,7-dimethoxy-*N*-(3-bromophenyl)-4-aminoquiazoline (IC_50_ 13.7 µM). Furthermore, one derivative exhibited higher activity than the reference drug against a stomach cancer cell line (IC50 values: 4.7 µM and 6.9 µM, respectively).

Interestingly, a significant influence of the absolute configuration of tested compounds on the antiproliferative activity was observed. Generally, d-isomers revealed higher growth inhibition in comparison with l-isomer derivatives [376]. To enhance antiproliferative activity, another amino acid residue (glycine or more rigid fragment, e.g., l-proline) was introduced into the pseudo-peptide scaffold [378]. This new series of chiral thiourea derivatives was examined toward BGC-823 (human gastric cancer) and A-549 (human non-small cell lung cancer) cell lines. The basic SAR studies led to the statement that the presence of the rigid l-proline fragment in the corresponding dipeptide structure increases antiproliferative activity. Moreover, antitumor properties may be improved by introducing an electron-withdrawing group in the *para* position of the terminal phenyl group of the dipeptide thioureas [377].

**Figure 41:**
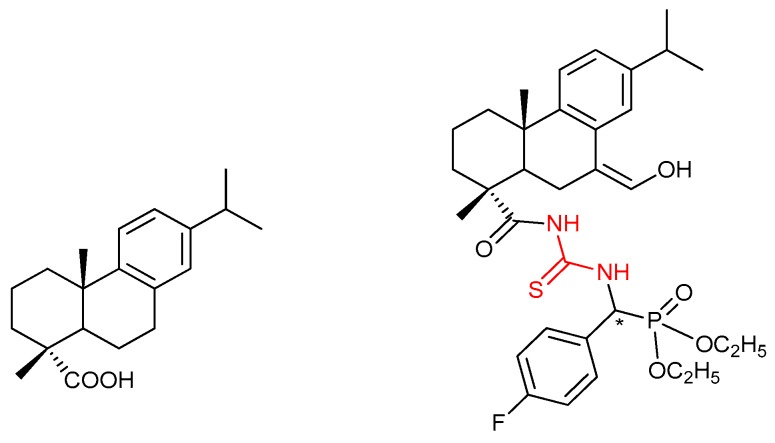
Dehydroabietic acid and its most active thiourea derivative [379].

Huang et al. demonstrated the results of anticancer activity assays of thioureas containing the α-aminophosphonate moiety based on dehydroabietic acid (DHA, Figure 41) skeleton [379]. It was found that the DHA increases antiproliferative action of drugs on various cancer cells [380]. Hence, a new series of thioureas incorporating the α-aminophosphonate moiety and DHA core was synthesized and tested in vitro against NCI-H460 (lung), A549 (lung adenocarcinoma), HepG2 (liver) and SKOV3 (ovarian) human cancer cell lines. The compounds exhibited moderate to high level of antiproliferative activity. The most active derivative revealed better results against A549 cell line than 5-fluorouracil, the medicine commonly used in cancer therapy (Figure 41). A preliminary analysis of mechanism of its action proved that the compound is capable to induce cell apoptosis [379].

**Figure 42:**
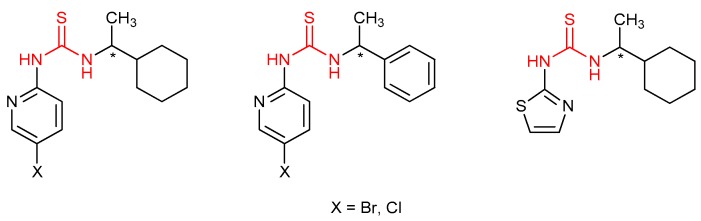
A new set of chiral halopyridyl and thiazolyl thioureas studied as antiproliferative agents [381].

The results of previous research on impact of stereochemistry of halopyridyl and thiazolyl thioureas on anti-HIV activity [372], prompted Venkatachalam’s group to extent studies toward anti-leukemic activity. They designed and synthesized five series of new chiral derivatives (Figure 42) [381]. Their anticancer activity was evaluated against human B-lineage Nalm-6 and T-lineage Molt-3 acute lymphoblastic leukemia cell lines. Preliminary studies proved that the stereochemistry indeed was the factor that determined the activity of tested compounds: (*S*) enantiomers performed better in the tests.

### 4.3. Anti-Allergic Thioureas

Venkatachalam et al. reported the synthesis and evaluation of anti-allergic activity of novel chiral heterocycle-based thioureas [382]. Referring to the fact that leukotrienes, chemical mediators released by mast cells, play an important role in pathophysiology of allergy and asthma, they became a new target for potential thiourea based anti-allergic agents [383].

**Figure 43:**
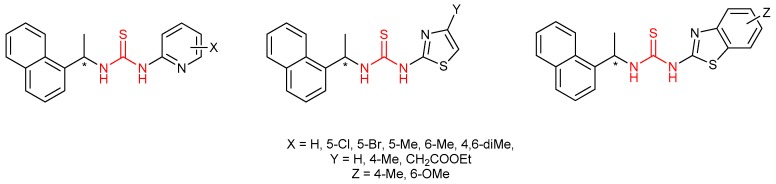
Chiral naphthyl-substituted thioureas as potential anti-allergic agents [383].

The set of indolyl-, naphthyl- and phenylethyl-substituted halopyridyl, thiazolyl and benzothiazolyl thiourea derivatives were tested in vitro for the mast cell inhibitory activity (Figure 43). Among them, naphthyl-substituted thiazolyl thioureas were found most promising. Based on the results obtained for (*S*)- and (*R*)-isomer of naphthyl thioureas it was concluded that the stereochemistry of studied thioureas did not greatly influence their activity.

### 4.4. Antimicrobial Thioureas

Pyrazole derivatives have been recognized as versatile compounds with multiple biological properties, e.g., antibacterial, anti-inflammatory or antiproliferative activity [384,385]. Many scientific groups have been involved in the investigation and development of novel pyrazole derivatives, facing the problem of their high toxicity.

**Figure 44:**
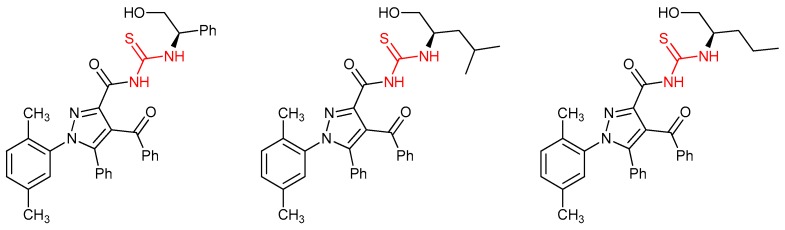
Novel chiral pyrazole-based thioureas exhibiting antibacterial activity [383].

The connection of thiourea functional group with these biologically active molecules led to a discovery of new compounds with a potential pharmaceutical importance. Bildirici and co-workers synthesized novel chiral pyrazole-based thioureas (Figure 44) [383]. The compounds were examined against three Gram-positive bacteria (*Bacillus subtilis*, *Staphylococcus aureus*, *Bacillus megaterium*) and four Gram-negative bacteria (*Enterobacter aerogenes*, *Pseudomonas aeruginosa, Klebsiella pneumoniae* and *Escherichia coli*) and exhibited a desired activity, one of them even higher than amikacin and rifampicin and similar to penicillin. Additionally, they were evaluated as antifungal agents against three fungal strains (*Candida albicans*, *Saccharomyces cerevisiae* and *Yarro*via *lipolytica*).

**Figure 45:**
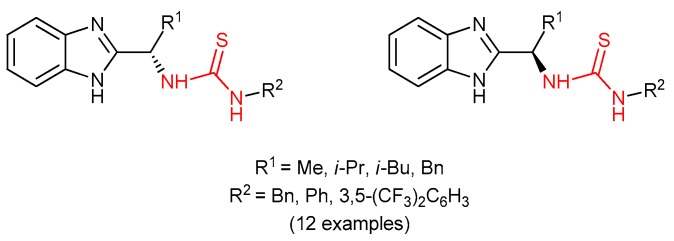
Novel chiral thioureas bearing benzimidazole fragment studied by Madabhushi et al. [388].

In the literature, there are several reports concerning thiourea functional group combined with heterobicyclic fused aromatic scaffolds, e.g., benzothiazole or benzimidazole rings [386,387]. Madabhushi et al. prepared two sets of chiral thioureas bearing benzimidazole ring based on natural and non-natural amino acids ((*S*)-alanine, (*S*)-phenylalanine, (*S*)-valine, (*S*)-leucine and (*R*)-alanine, (*R*)-phenylalanine, (*R*)-valine and (*R*)-leucine, respectively; Figure 45) [388]. The obtained compounds were studied as potential antimicrobial agents against the Gram-positive strains *Staphylococcus aureus*, *Bacillus subtilis*, *Staphylococcus aureus* and *Micrococcus luteus* as well as the Gram-negative strains *Klebsiella planticola*, *Escherichia coli* and *Pseudomonas aeruginosa*. Additionally, antiproliferative activity was examined against A549, MCF7, DU145 and HeLa cell lines.

Interestingly, the compounds derived from natural amino acids exhibited antibacterial activity, whereas their isomers turned out to be inactive against the tested Gram-positive and Gram-negative strains. Thioureas substituted with isopropyl or isobutyl and 3,5-(CF_3_)_2_C_6_H_3_ groups were the most potent among the series. It was considered that a suitable stereochemistry and the presence of lipophilic trifluoromethyl groups which may increase bioavailability and bio-efficiency, were the factors that determined antibacterial activity.

**Figure 46:**
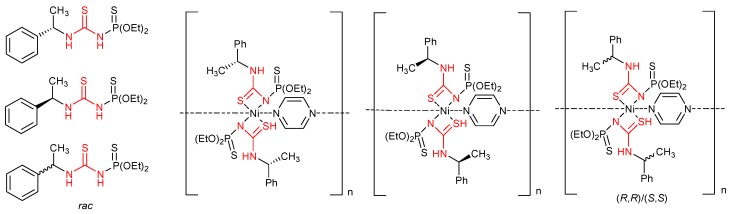
Structures of thiophosphorylated thioureas and their Ni(II) complexes forming coordination polymers studied for their antimicrobial activity [22].

Chiral thiophosphorylated thioureas have attracted an attention due to their biological activity, and ability to form diverse complexes with transition metals. Based on literature reports Metlushka et al. synthesized new coordination polymers with chiral thiophosphorylated thioureas in both racemic and enantiopure forms and checked their antimicrobial activity against *S. aureus* and *B. cereus* [22]. They examined both enantiopure and racemic derivatives as well as their complexes with Ni(II) (Figure 46). Neither (*R*)- nor (*S*)-ligands did exhibit valuable anti-microbial activity, while the racemic mixture was surprisingly active. In case of complexes, (*R*)-complex was less active than (*S*)-complex against *S. aureus* while the racemate probably was not considered in the research. In turn, both isomers and the racemic mixture performed similarly against *B. cereus.*

## 5. Summary

A relatively simple, rigid skeleton of thiourea can be combined with chiral moieties, yielding a system that is capable of strong and selective interactions with a variety of chiral molecules, including compounds of biological importance. By an appropriate substitution we can suitably modify their properties, and, in principle, there are no limitations in preparation of desired mono-, di-, tri- or tetrasubstituted, aliphatic or aromatic, unsymmetrical, or symmetrical thiocarbamides. Recently published synthetic methods extend the palette of possible reactants, and often focus on the modification of conditions: the reaction can be performed in water, with ultrasound-assistance, and even without any solvent and catalyst.

Chiral thioureas have already proven their utility in various stereoselective reactions, mainly as efficient organocatalysts and chiral ligands. As shown by numerous examples, a proper choice of a chiral component present in the structure of thiourea and its configuration can also result in a desired biological activity. This is manifested in a considerable interest in the use of these compounds as pharmaceuticals and in agriculture, and biomedical applications of chiral thioureas should become an important and growing area.
